# KIR-HLA and Maternal-Infant HIV-1 Transmission in Sub-Saharan Africa

**DOI:** 10.1371/journal.pone.0016541

**Published:** 2011-02-04

**Authors:** Maria Paximadis, Gregory Minevich, Robert Winchester, Diana B. Schramm, Glenda E. Gray, Gayle G. Sherman, Ashraf H. Coovadia, Louise Kuhn, Caroline T. Tiemessen

**Affiliations:** 1 AIDS Virus Research Unit, National Institute for Communicable Diseases, Johannesburg, South Africa; 2 Department of Medicine, College of Physicians and Surgeons, Columbia University, New York, New York, United States of America; 3 Perinatal HIV Research Unit, Chris Hani Baragwanath Hospital, Soweto, South Africa; 4 Department of Molecular Medicine and Haematology, University of the Witwatersrand Medical School, Johannesburg, South Africa; 5 National Health Laboratory Services, Johannesburg, South Africa; 6 Empilweni Clinic, Coronation Women and Children Hospital, Enhancing Childhood HIV Outcomes (ECHO), University of the Witwatersrand, Johannesburg, South Africa; 7 Gertrude H. Sergievsky Centre, College of Physicians and Surgeons and Department of Epidemiology, Mailman School of Public Health, Columbia University, New York, New York, United States of America; 8 Faculty of Health Sciences, University of the Witwatersrand, Johannesburg, South Africa; Karolinska Institutet, Sweden

## Abstract

Numerous studies have suggested a role for natural killer (NK) cells in attenuation of HIV-1 disease progression via recognition by killer-cell immunoglobulin-like receptors (KIRs) of specific HLA class I molecules. The role of KIR and HLA class I has not been addressed in the context of maternal-infant HIV-1 transmission. *KIR* and *HLA class I B* and *C* genes from 224 HIV-1-infected mothers and 222 infants (72 infected and 150 uninfected) from South Africa were characterized. Although a number of significant associations were determined in both the total group and in the nevirapine (NVP) exposed group, the most significant findings involved *KIR2DL2* and *KIR2DL3* and HLA-C. *KIR2DL2*/*KIR2DL3* was underrepresented in intrapartum (IP)-transmitting mothers compared to non-transmitting (NT) mothers (*P* = 0.008) and remained significant (*P* = 0.036) after correction for maternal viral load (MVL). Homozygosity for *KIR2DL3* alone and in combination with HLA-C allotype heterozygosity (*C1C2*) was elevated in IP-transmitting mothers compared to NT mothers (*P* = 0.034 and *P* = 0.01 respectively), and after MVL correction (*P* = 0.033 and *P* = 0.027, respectively). In infants, *KIR2DL3* in combination with its HLA-C1 ligand (C1) as well as homozygosity for *KIR2DL3* with *C1C2*, were both found to be underrepresented in infected infants compared to exposed uninfected infants in the total group (*P* = 0.06 and *P* = 0.038, respectively) and in the sub-group of infants whose mothers received NVP (*P* = 0.007 and *P* = 0.03, respectively). These associations were stronger post MVL adjustment (total group: *P* = 0.02 and *P* = 0.009, respectively; NVP group: *P* = 0.004 and *P* = 0.02, respectively). Upon stratification according to low and high MVL, all significant associations fell within the low MVL group, suggesting that with low viral load, the effects of genotype can be more easily detected. In conclusion this study has identified a number of significant associations that suggest an important role for NK cells in maternal-to-infant HIV-1 transmission.

## Introduction

Natural killer (NK) cells, large bone marrow-derived granular lymphocytes, are classically defined as playing an integral role in the innate immune response by targeting virally-infected cells and transformed cells with direct killing. In addition, they are known to be involved in the adaptive immune response by providing help through cytokine secretion. Increasingly however, the role of NK cells in viral control is looking more complex. A mouse model of cytomegalovirus infection has shown NK cells to display ‘memory’ features typical of an adaptive immune response [1]. NK cells have also been shown to respond with marked specificity to HIV-1 peptides as measured by a whole blood intracellular cytokine assay [2], [3], [4].

NK cell function is facilitated by a repertoire of receptors that are encoded by several gene families, amongst which are the killer immunoglobulin(Ig)-like receptor (KIR) genes that are located on chromosome 19q13.4 and encode both activating and inhibitory KIR receptors (see review: [5]). Expression of KIR receptors is complex and controlled by a stochastic mechanism that shuts off expression of some receptors and not others in individual cells thereby allowing different NK cell clones to recognize their targets differentially (see review: [6]). In addition, there is a high degree of polymorphism in the *KIR* gene family and differences among individuals with regard to *KIR* gene haplotypes that vary in number and types of genes present [7], [8]. The KIR receptors are type I integral membrane glycoproteins that are usually expressed on the cell surface as monomers and 14 distinct *KIR* genes and two pseudogenes have been described to date (see http://www.ebi.ac.uk/kir for latest updates on *KIR* genes and alleles). The KIR receptors are named according to the number (i.e. 2 or 3) of Ig-like domains present in the extracellular region as well as the length (i.e. L: long or S: short) of their cytoplasmic tails. KIR receptors can be divided into two broad groups based on function i.e. either inhibitory [characterized by L cytoplasmic domains with immunoreceptor tyrosine-based inhibition motifs (ITIMs) in these domains] or activating [characterized by S cytoplasmic domains with immunoreceptor tyrosine-based activation motifs (ITAMs) in these domains] (see review: [9]). The ligands for KIR receptors for the most part are the HLA class I molecules [10].

Increasingly, the part that NK cells play in the control of HIV-1 infection is being investigated with many genetic association studies of the role of select KIR receptors in isolation, or in combination with their HLA ligands, on HIV-1 disease progression and to a more limited extent HIV-1 adult transmission [11], [12], [13], [14], [15], [16], [17], [18], [19]. Although these genetic association studies suggest a role for KIR-HLA interactions in the control of HIV infection, they tend to disagree on the exact nature of these associations. The KIR receptors that seem to be consistently highlighted as key players however, are KIR3DS1, KIR3DL1 and to some extent KIR2DL3. These studies have mostly addressed associations with disease progression among individuals who are already infected with HIV and the role of these genotypes in protection against infection in the first place has not been established. These studies also mostly include populations in the U.S. and Europe and have not as extensively interrogated associations among sub-Saharan African populations who are the people most affected by the HIV epidemic. In recent studies conducted in our laboratory, unusual robust NK cell responses to HIV-1 peptides were significantly associated with reduced maternal-infant HIV-1 transmission and were also associated with control of HIV-infection in the mothers [3], [4]. The role of KIR and HLA in the context of maternal-infant HIV-1 transmission has not been previously investigated. Here we describe the *KIR* and *HLA* gene content among several mother-infant HIV-1 cohorts, in order to investigate if *KIR* genes and/or *KIR* genes together with their known HLA ligands may play a role in susceptibility of infants to HIV-1 infection and/or in modulating the risk of maternal HIV-1 transmission to the infant. Significant KIR-HLA associations identified in the current study, particularly involving *KIR2DL2*, *KIR2DL3* and *HLA-C*, support a role for NK cells in both maternal HIV-1 transmissibility and infant HIV-1 susceptibility.

## Results

### KIR genes/haplotypes/genotypes

#### KIR genes

All 14 *KIR* genes and the two pseudogenes were determined in both the mother and infant groups. The frequencies of the individual *KIR* genes/pseudogenes in the various subgroups within the mothers and infants, as well as the frequency comparisons between groups, are shown in supplementary Tables S1 and S2 respectively. There were no significant differences in comparisons of these frequencies within the mothers or infant groups. The frequencies of the respective genes found in this cohort were well within the range of frequencies reported for other sub-Saharan African countries [20]. Comparison of gene number (total, activating and inhibitory) and gene ratio (inhibitory to activating genes) (Table 1) revealed IU-transmitting mothers to have a trend towards lower total gene numbers compared to NT mothers (*P* = 0.06). This seemed to be largely contributed by the inhibitory *KIR* genes, since IU-transmitting mothers had a significantly lower number of inhibitory genes compared to NT mothers (*P* = 0.04). These two associations were significant post maternal viral load (MVL) adjustment (gene number: *P* = 0.027; OR = 0.73, inhibitory genes: *P* = 0.033; OR = 0.51) but did not seem to be influenced by stratification into low and high MVL groups (Table 2). Infants showed no significant differences in gene number and ratio (Table 1).

**Table 1:** Mann-Whitney U test comparisons of *KIR* gene numbers and ratios in the mother and infant groups.

Mann-Whitney *U* test comparison	Mothers						
	Median (range)				*P*		
	TR	IP	IU	NT	TR vs NT	IP vs NT	IU vs NT
Total gene number	12 (7–16)	12 (9–16)	10 (7–14)	12 (9–16)	0.72	0.87	**0.06**
Activating genes	3 (1–6)	3 (1–6)	2 (1–5)	3 (1–6)	0.61	0.63	0.15
Inhibitory genes	7 (4–8)	7 (6–8)	6 (4–8)	7 (6–8)	0.12	0.19	**0.04**
Ratio: inhibitory to activating genes	2.7 (1.2–6)	2.7 (1.2–6)	3.5 (1.3–6)	2.7 (1.2–6)	0.39	0.42	0.27

Mann-Whitney *U* test comparison	Infants						
	Median (range)				*P*		
	INF	IP	IU	EU	INF vs EU	IP vs EU	IU vs EU
Total gene number	12 (7–16)	11.5 (7–14)	12 (9–16)	12 (8–16)	0.74	0.76	0.54
Activating genes	3 (1–6)	2 (1–4)	3 (1–6)	3 (1–6)	0.60	0.51	0.98
Inhibitory genes	7 (5–8)	7 (5–8)	7 (5–8)	7 (5–8)	0.94	0.92	0.43
Ratio: inhibitory to activating genes	2.7 (1.2–7)	3.3 (1.8–7)	2.7 (1.3–6)	2.7 (1.2–6)	0.59	0.37	0.79

Bold *P* values indicate trends (0.05<*P*<0.1) or significant differences (*P*<0.05).

**Table 2:** Summary of all key associations with adjustments made for maternal viral load.

Genotypic feature	Relationship between groups		Deleterious (D) or Advantageous (A)	OR	*P*	MVL Adjusted OR	MVL Adjusted*P*	OR if MVL low	OR ifMVL high	*P* if significant*
**Total group comparisons**										
**Mothers**										
Total *KIR* gene Number	IU < NT		A	0.76^a^	0.03 a	0.73	0.027	0.68	0.77	
*KIR* inhibitory genes	IU < NT		A	0.54^a^	0.02 a	0.51	0.033	0.52	0.56	
Bx20 *KIR* genotype	TR < NT		A	0.12	0.02	0.17	0.09	UD	0.385	
*KIR2DL2*/*KIR2DL3*	TR < NT		A	0.53	0.044	0.55	0.066	0.31*	0.74	0.04
*KIR2DL2*/*KIR2DL3*	IP < NT		A	0.29	0.008	0.32	0.036	0.133	0.43	
Bx32 *KIR* genotype	TR > NT		D	8.50	0.04	8.98	0.097	UD	2.45	
Bx32 *KIR* genotype	IP > NT		D	17.2	0.014	27.3	0.011	UD	3.38	
*KIR2DL3*/*KIR2DL3*	IP > NT		D	2.42	0.034	2.70	0.033	6.72*	1.71	0.03
*KIR2DS2*+*C1C1*	TR > NT		D	2.45	0.074	1.79	0.293	6.97*	0.996	0.04
*KIR2DL2*/*KIR2DL2*+*C1C1*	TR > NT		D	5.36	0.041	3.55	0.178	UD	1.85	
*KIR2DL3*/*KIR2DL3*+*C1C2*	IP > NT		D	3.63	0.010	3.26	0.027	11.83*	1.26	0.002
**Infants**										
*KIR3DL1*+*Bw480I*	IP < EU		A	0.41	0.062	0.51	0.17	0.17	0.75	
*KIR2DL2*/*KIR2DL2*	IP < EU		A	0.16	0.052	0.13	0.058	UD	0.24	
*KIR2DL3*+*C1*	INF < EU		A	0.58	0.062	0.47	0.02	0.30*	0.59	0.03
*KIR2DL3*/*KIR2DL3*+*C1C2*	INF < EU		A	0.40	0.038	0.25	0.009	0.19	0.32	
*KIR2DL3*/*KIR2DL3*+*C2C2*	IP > EU		D	2.87	0.086	5.57	0.008	5.18*	3.64	0.05
**NVP stratified group comparisons**										
**Mothers**										
Bx20 *KIR* genotype	TR < NT:	MNVP	A	0.00	0.03	UD	UD	UD	UD	
*KIR2DL2*/*KIR2DL3*	TR < NT:	MNVP	A	0.49	0.08	0.40	0.039	0.18*	0.73	0.04
*KIR2DL2*/*KIR2DL3*	IP < NT:	MNVP	A	0.14	0.04	0.14	0.08	0.27	UD	
Bx32 *KIR* genotype	TR > NT:	noMNVP	D	7.29	0.09	15.4	0.046	UD	1.22	
*KIR2DL3*/*KIR2DL3*+*C2C2*	TR > NT:	MNVP	D	2.55	0.07	2.49	0.074	2.93	2.45	
**Infants**										
*KIR2DL3*/*KIR2DL3*+*C1C2*	INF < EU:	MNVP	A	0.29	0.03	0.22	0.021	0.24	0.19	
*KIR2DL3*+*C1*	INF < EU:	MNVP	A	0.32	0.007	0.29	0.004	0.15*	0.52	0.007
*KIR2DL3*/*KIR2DL3*+*C2C2*	INF > EU:	noMNVP	D	5.10	0.07	6.16	0.072	23.8*	1.91	0.03
*KIR2DL2*/*KIR2DL2*	INF > EU:	MNVP	D	2.33	0.08	1.90	0.211	4.54*	0.98	0.06
*KIR2DL2*/*KIR2DL2*+*C1C2*	INF > EU:	MNVP	D	4.46	0.06	3.51	0.116	8.00	2.05	
*KIR2DL3*/*KIR2DL3*+*C2C2*	INF > EU:	MNVP	D	2.21	0.16	3.64	0.033	2.61	3.54	
*KIR2DL3*/*KIR2DL3*+*C2C2*	IP > EU:	noMNVP	D	8.50	0.02	10.42	0.02	48*	4.2	0.02

a : Odds Ratios and *P* values are from logistic regression analysis; UD: undetermined due to insufficient data points.

MVL: Maternal viral load; NVP: Nevirapine; MNVP: Maternal nevirapine; noMNVP: No maternal nevirapine.

NT: Non-transmitting TR: Transmitting; INF: Infected; EU: Exposed uninfected; IP: Intrapartum; IU: Intrauterine.

In the NVP-stratified group comparisons, no significant associations were seen in either mothers and infants when individual *KIR* genes, or *KIR* gene number (total, inhibitory, activating) and ratios (inhibitory:acitivating) were compared (data not shown).

#### Haplotypes/Genotypes

**Figure 1:**
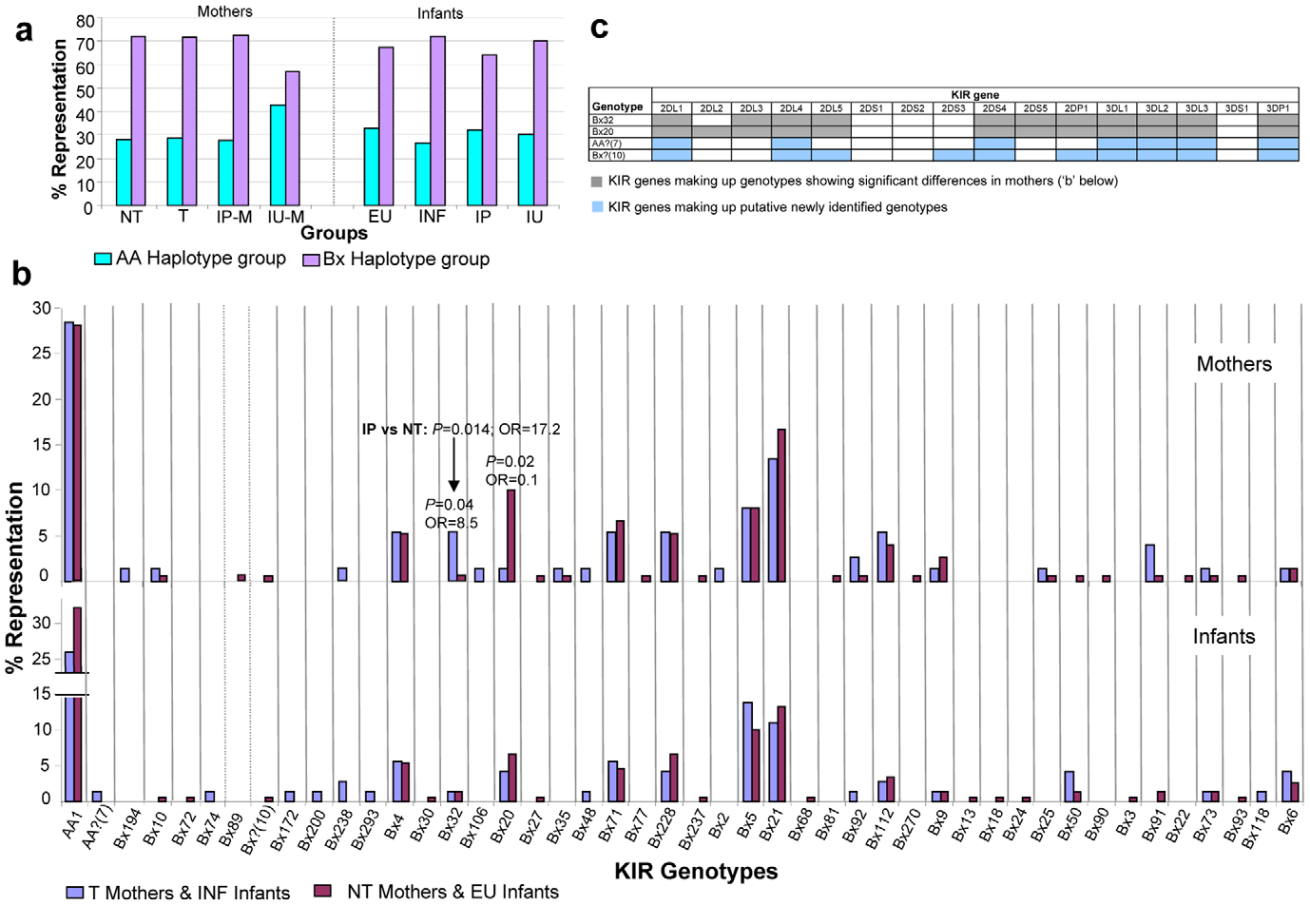
*KIR* gene haplotypes and genotypes. **a**: *KIR* gene haplotype (AA and Bx) representation in mother and infant groups. **b**: *KIR* genotype representation in mothers and infants. *P* values for the significant differences between groups are shown. **c**: Individual *KIR* genes making up the two genotypes (Bx20 and Bx32) showing significance in mothers (b) and *KIR* genes involved in the two putative newly identified genotypes in the current study population [designated as AA?(7) and Bx?(10)].

Representation of AA and Bx haplotypes within the mother and infant groups can be seen in Figure 1a. The ratio of AA haplotype:Bx haplotype groups ranged from 1∶2 to 1∶2.6 across groups (i.e. at least double Bx haplotype representation compared to the AA haplotype), except for the IU-transmitting mothers, where the ratio of AA:Bx haplotype groups was 1∶1.3. Comparisons of IU and NT mothers with respect to AA and Bx haplotype groups, however, were not significant (ORs of 1.93 and 0.52 respectively, *P* = 0.203 for both). Composition of the broader haplotype groups with respect to *KIR* gene content (genotypes) for the mother and infant groups is shown in Figure 1b. Although the IP and IU mother and corresponding infant groups were also compared, only the broader groups are depicted in Figure 1b. Thirty-three different genotypes were detected in the mothers and 35 in the infant group, with a total of 46 different genotypes detected in the combined mother-infant group. Two previously unreported (http://www.allelefrequencies.net [20]) genotypes comprised of 7 (AA?) and 10 (Bx?) *KIR* genes were detected in a single INF infant, and an NT mother and EU infant respectively. The genes involved in these two ‘new’ *KIR* genotypes are shown in Figure 1c. These ‘new’ genotypes are only putative at this stage and would need to be verified in order for official acceptance as new genotypes. The AA1 genotype was the most highly represented genotype in both the mother and infant groups. The Bx genotypes, Bx5 and Bx21, were the most highly represented genotypes in both the mother and infant groups.

Transmitting mothers had significantly lower representation of Bx20 compared to NT mothers (1.35% vs. 10%; *P* = 0.02; OR = 0.1) and TR mothers had significantly higher representation of Bx32 compared to NT mothers (5.4% vs. 0.7%; *P* = 0.04; OR = 8.5). This relationship was more pronounced when IP mothers were compared to NT mothers where IP mothers had 10.3% representation of Bx32 compared to the 0.7% in NT mothers (*P* = 0.014; OR = 17.2). Upon correction for MVL, Bx20 only showed a trend (*P* = 0.09; OR = 0.17) of lower representation in TR mothers vs. NT mothers (Table 2), however maternal nevirapine (NVP) stratification (Table 3) revealed Bx20 to maintain significant lower representation in TR mothers vs. NT mothers in the group of mothers that received single dose NVP (*P* = 0.03; OR = 0.00). Bx32 maintained significant high representation in IP mothers vs NT mothers post MVL correction (Table 2: *P* = 0.01; OR = 27.3) and although only a trend (*P* = 0.09; OR = 7.29) of high representation in TR mothers vs. NT mothers was seen upon stratification in the group of mothers that did not receive NVP (noMNVP) (Table 3), this was significant post MVL correction for this subgroup (Table 2: *P* = 0.046; OR = 15.4). The genes making up genotypes Bx32 and Bx20 are also shown in Figure 1c. No significant differences with respect to *KIR* genotypes were seen in the infants.

**Table 3:** Summary of key associations in mother and infant groups according to maternal single dose nevirapine.

Key associations	Association^a^ in overall group	Association^a^ in NVP- stratified group	No maternal NVP			Maternal NVP		
**Mothers**			**OR**	**95% CI**	***P***	**OR**	**95% CI**	***P***
			**TR (24) vs NT (52)**			**TR (42) vs NT (82)**		
*KIR2DL3*/*KIR2DL3* + *C2C2*	**NS**	**T:** TR > NT (D)	1.48	0.23–9.52	0.65	2.55	0.98–6.63	**0.07**
Bx20 *KIR* genotype	**S:** TR < NT (A)	**S:** SA^b^	0.40	0.05–3.70	0.66	0.00	0.00-NaN	**0.03**
*KIR2DL2*/*KIR2DL3*	**S:** TR < NT (A)	**T:** SA	0.55	0.21–1.50	0.32	0.49	0.22–1.08	**0.08**
Bx32 *KIR* genotype	**S:** TR > NT (D)	**T:** SA	7.29	0.72–74.11	**0.09**	∞	NaN-∞	0.34
*KIR2DL2*/*KIR2DL2* + *C1C1*	**S:** TR > NT (D)	-	∞	NaN-∞	0.32	3.08	0.49–19.18	0.34
			**IP (16) vs NT (52)**			**IP (10) vs NT (82)**		
*KIR2DL2/KIR2DL3*	**S:** IP < NT (A)	**S:** SA	0.42	0.13–1.38	0.17	0.14	0.02–1.12	**0.04**
*KIR2DL3/KIR2DL3*	**S:** IP > NT (D)	-	2.25	0.72–7.06	0.23	2.41	0.64–9.12	0.28
*KIR2DL3/KIR2DL3* + *C1C2*	**S:** IP > NT (D)	-	2.52	0.74–8.58	0.18	2.31	0.42–12.82	0.30
**Infants**			**OR**	**95% CI**	***P***	**OR**	**95% CI**	***P***
			**INF (24) vs EU (53)**			**INF (41) vs EU (81)**		
*KIR2DL2*/*KIR2DL2* + *C1C2*	**NS**	**T:** INF > EU (D)	0.29	0.03–2.46	0.42	4.46	1.05–18.85	**0.06**
*KIR2DL3*/*KIR2DL3* + *C1C2*	**S:** INF < EU (A)	**S:** SA	0.51	0.10–2.61	0.72	0.29	0.09–0.91	**0.03**
*KIR2DL3* + *C1*	**T:** INF < EU (A)	**S:** SA	0.85	0.32–2.27	0.80	0.32	0.15–0.71	**0.007**
*KIR2DL2*/*KIR2DL2*	**T:** IP < EU (A)	**T:** INF > EU (D)	0.25	0.05–1.22	0.13	2.33	0.91–5.97	**0.08**
*KIR2DL3*/*KIR2DL3* + *C2C2*	**T:** IP > EU (D)	**T:** INF > EU (D)	5.10	0.86–30.07	**0.07**	2.21	0.76–6.40	0.16
			**IP (16) vs EU (53)**			**IP (10) vs EU (81)**		
*KIR2DL2*/*KIR2DL2*	**T:** IP < EU (A)	-	0.19	0.02–1.54	0.16	0.00	0.00- NaN	0.60
*KIR2DL3*/*KIR2DL3* + *C2C2*	**T:** IP > EU (D)	**S:** SA	8.50	1.39–51.95	**0.02**	2.28	0.41–12.65	0.30

a S: significant association (*P*<0.05); T: trend (0.05<*P*<0.1); NS: Not significant; A: Advantageous; D: Deleterious.

b SA: Same association i.e. the trend or significant association is going in the same direction as non-stratified group comparison.

**-:** Not applicable (i.e. not significant or no trend in stratified groups).

### KIR-HLA associations

#### Hardy-Weinberg Equilibrium

No significant deviations from Hardy-Weinberg proportions were noted for either *HLA-B* or *HLA-C* in both the infant and mother groups (all *P*'s>0.1).

#### KIR-HLA-B

Since *KIR3DL1* and *KIR3DS1* segregate as alleles of the same locus and are both thought to encode for receptors that bind HLA-Bw4 ligands, comparisons across the mother and infant groups involved looking at the dose of *KIR* and *Bw* alleles independently as well as various combinations of *KIR-HLA.* The results of these comparisons in the mothers and infants can be found in Supplementary Tables S3 and S4, respectively. No significant differences were seen with either the mother or the infant groups with respect to these alleles and *KIR-HLA* combinations. A trend however was noted in the infant group where IP infants had approximately 20% lower representation of *KIR3DL1*+*Bw480Ile* compared to EU infants (*P* = 0.062; OR = 0.41) (Table S4), however this trend was lost post MVL adjustment (Table 2) and upon NVP stratification (data not shown).

#### KIR-HLA-C: Mothers

Similarly, as for *KIR3DL1* and *KIR3DS1*, *KIR2DL2* and *KIR2DL3* segregate as alleles of the same locus, thus comparisons involved looking at the allelic dose of these genes and their respective *HLA-C* allotypes as well as the *KIR-HLA* combinations. Results for the overall group mother comparisons are shown in Table 4.

**Table 4:** Comparison of frequencies of *KIR2DL2*, *KIR2DL3* and *HLA-C* allotypes as well as *KIR-HLA-C* combinations between HIV-1-transmitting (TR) mothers, intrapartum (IP)-HIV-1-transmitting mothers, intrauterine (IU)-HIV-1 transmitting mothers and non-transmitting (NT) mothers.

Genetic factor	TR mothers(N = 74)	IP mothers (N = 29)	IUmothers(N = 21)	NTmothers (N = 150)	T mothers vs NT mothers			IP mothers vs NT mothers			IU mothers vs NT mothers		
	% representation				OR	95% CI	*P*	OR	95% CI	*P*	OR	95% CI	*P*
***KIR*** ** alleles**													
*2DL2/2DL2*	27.0	27.6	19.1	21.3	1.37	0.72–2.60	0.401	1.40	0.57–3.47	0.470	0.87	0.27–2.76	1.000
*2DL2/2DL3*	32.4	20.7	33.3	47.3	0.53	0.30–0.96	**0.044**	0.29	0.11–0.75	**0.008**	0.56	0.21–1.46	0.252
*2DL3/2DL3*	39.2	51.7	42.9	30.7	1.46	0.81–2.61	0.230	2.42	1.08–5.43	**0.034**	1.70	0.67–4.30	0.319
***HLA-C*** ** alleles**													
*C1/C1*	16.2	3.5	23.8	14.0	1.19	0.55–2.57	0.691	0.22	0.03–1.70	0.211	1.92	0.64–5.80	0.325
*C1/C2*	48.7	62.1	47.6	50.0	0.95	0.54–1.65	0.888	1.64	0.72–3.70	0.310	0.91	0.36–2.27	1.000
*C2/C2*	35.1	34.5	28.6	36.0	0.96	0.54–1.72	1.000	0.94	0.41–2.16	1.000	0.71	0.26–1.94	0.628
***KIR-HLA*** ** combinations**													
*2DL1*+*C2*	82.4	96.6	71.4	85.3	0.81	0.38–1.71	0.564	4.81	0.62–37.21	0.132	0.43	0.15–1.23	0.119
*2DL2*+*C1*	41.9	31.0	47.6	44.7	0.89	0.51–1.57	0.775	0.56	0.24–1.30	0.219	1.13	0.45–2.81	0.819
*2DL3*+*C1*	44.6	48.3	47.6	50.0	0.80	0.46–1.41	0.479	0.93	0.42–2.07	1.000	0.91	0.36–2.27	1.000
*2DS1*+*C2*	12.2	20.7	5.0	9.3	1.35	0.55–3.27	0.494	2.53	0.88–7.27	0.102	0.49	0.06–3.90	0.697
*2DS2*+*C1*	43.2	34.5	47.6	36.0	1.35	0.77–2.39	0.310	0.94	0.41–2.16	1.000	1.62	0.64–4.05	0.340
*2DL1*+*C2C2*	35.1	34.5	28.6	36.0	0.96	0.54–1.72	1.000	0.94	0.41–2.16	1.000	0.71	0.26–1.94	0.628
*2DL2*+*C1C1*	13.5	3.5	14.3	7.3	1.97	0.80–4.89	0.149	0.45	0.06–3.64	0.694	2.11	0.54–8.27	0.385
*2DL3*+*C1C1*	9.5	3.5	19.1	12.7	0.72	0.29–1.80	0.658	0.25	0.03–1.92	0.205	1.62	0.49–5.34	0.491
*2DS1*+*C2C2*	4.1	6.9	0.0	4.0	1.01	0.25–4.17	1.000	0.56	0.11–2.92	0.617	0.00	0.00–NaN	1.000
*2DS2*+*C1C1*	13.5	3.5	14.3	6.0	2.45	0.95–6.32	**0.074**	1.77	0.22–14.57	1.000	2.61	0.65–10.54	0.169
*2DL2/2DL2*+*C1C1*	6.8	0.0	4.8	1.3	5.36	1.01–28.33	**0.041**	0.00	0.00-NaN	1.000	3.70	0.32–42.68	0.327
*2DL2/2DL2*+*C2C2*	8.1	10.3	0.0	7.3	1.12	0.40–3.14	0.795	1.46	0.38–5.59	0.703	0.00	0.00-NaN	0.363
*2DL2/2DL2*+*C1C2*	12.2	17.2	14.3	12.7	0.95	0.41–2.23	1.000	1.44	0.49–4.22	0.552	1.15	0.31–4.27	0.737
*2DL3/2DL3*+*C1C1*	2.7	0.0	9.5	7.3	0.39	0.08–1.82	0.345	0.00	0.00-NaN	0.370	1.47	0.30–7.24	0.644
*2DL3/2DL3*+*C2C2*	17.6	17.2	23.8	11.3	1.64	0.75–3.59	0.219	1.63	0.55–4.84	0.363	2.44	0.79–7.52	0.155
*2DL3/2DL3*+*C1C2*	18.9	34.5	9.5	12.7	1.61	0.76–3.42	0.233	3.63	1.47–8.96	**0.010**	0.73	0.16–3.37	1.000
*2DL2/2DL3*+*C1C1*	6.8	3.5	9.5	6.0	1.14	0.37–3.52	0.778	0.56	0.07–4.60	1.000	1.65	0.33–8.21	0.628
*2DL2/2DL3*+*C2C2*	9.5	6.9	4.8	16.7	0.52	0.21–1.27	0.162	0.37	0.08–1.66	0.259	0.25	0.03–1.95	0.206
*2DL2/2DL3*+*C1C2*	16.2	10.3	19.1	24.7	0.59	0.29–1.22	0.172	0.35	0.10–1.23	0.142	0.72	0.23–2.27	0.786

Bold *P* values indicate trends (0.05<*P*<0.1) or significant differences (*P*<0.05).

#### *KIR2DL2/KIR2DL3*

TR mothers had significantly lower representation of *KIR2DL2/KIR2DL3* than NT mothers (*P* = 0.044; OR = 0.53), which seems to be largely contributed by the IP transmitting mothers that have considerably less representation of this *KIR* allele combination compared to NT mothers (*P* = 0.008; OR = 0.29). TR mothers maintained this significant lower representation of *KIR2DL2/KIR2DL3* only in the context of low MVL (Table 2: *P* = 0.04; OR = 0.31), and showed a trend in the overall MVL adjustment (Table 2: *P* = 0.066; OR = 0.55) while IP mothers maintained significance post MVL correction (Table 2: *P* = 0.036; OR = 0.32). Stratification according to NVP showed the TR and IP mothers that received single dose NVP to have a trend (*P* = 0.08; OR = 0.49) and significant (*P* = 0.04; OR = 0.14) lower representation of *KIR2DL2/KIR2DL3* compared to NT mothers, respectively (Table 3), and, when these subgroups were corrected for MVL (Table 2), TR mothers maintained significant lower representation (*P* = 0.039; OR = 0.40) and also when stratified into the low MVL group (*P* = 0.04; OR = 0.18), while IP mothers only showed a trend post MVL correction (*P* = 0.08; OR = 0.14).

#### *KIR2DL3/KIR2DL3* and *KIR2DL3/KIR2DL3+C1C2*

IP mothers had significantly higher representation of homozygous *KIR2DL3* compared to NT mothers (*P* = 0.034; OR = 2.42). Furthermore, homozygosity for *KIR2DL3* in combination with heterozygosity for HLA-C allotype group (*C1C2*) was also present in IP mothers in significantly greater proportion compared to NT mothers (*P* = 0.01; OR = 3.63). Although both these significant associations were lost upon stratification according to NVP dose (Table 3), correction for MVL in the overall groups revealed that both remained significant post correction (Table 2: *KIR2DL3*/*KIR2DL3 P* = 0.033; OR = 2.70, *KIR2DL3*/*KIR2DL3*+*C1C2 P* = 0.027; OR = 3.26) and furthermore stratification into low and high MVL groups showed both to be significant in the context of low MVL (Table 2: *KIR2DL3*/*KIR2DL3 P* = 0.03; OR = 6.72, *KIR2DL3*/*KIR2DL3*+*C1C2 P* = 0.002; OR = 11.83).

#### *KIR2DL2/KIR2DL2+C1C1*

Homozygosity for *KIR2DL2* plus homozygosity for its ligand (*C1C1*) was significantly elevated in TR mothers compared to NT mothers (*P* = 0.041; OR = 5.36), this significance was however lost upon NVP stratification (Table 3) and post MVL adjustment (Table 2).

#### *KIR2DS2+C1C1*

The trend (*P* = 0.074; OR = 2.45) of higher representation of *KIR2DS2* together with homozygosity for its ligand (*C1C1*) in TR mothers compared to NT mothers was not present post NVP stratification or MVL adjustment, but did prove to be significantly elevated in TR mothers in the context of low MVL (Table 2: *P* = 0.04; OR = 6.97).

#### KIR-HLA-C: Infants

Results for the overall group infant comparisons are shown in Table 5.

**Table 5:** Comparison of frequencies of *KIR2DL2*, *KIR2DL3* and *HLA-C* allotypes as well as *KIR-HLA-C* combinations between HIV-1-infected (INF) infants, intrapartum (IP)-HIV-1 infected infants, intrauterine (IU)-HIV-1 infected infants and exposed uninfected (EU) infants.

Genetic factor	INFinfants(N = 72)	IPinfants(N = 28)	IUinfants(N = 20)	EUinfants (N = 150)	INF infants vs EU infants			IP infants vs EU infants			IU infants vs EU infants		
	% representation				OR	95% CI	*P*	OR	95% CI	*P*	OR	95% CI	*P*
***KIR*** ** alleles**													
*2DL2/2DL2*	19.4	3.6	30.0	18.7	1.05	0.52–2.15	1.000	0.16	0.02–1.24	**0.052**	1.87	0.66–5.29	0.241
*2DL2/2DL3*	45.8	50.0	35.0	44.7	1.05	0.60–1.84	0.886	1.24	0.55–2.78	0.681	0.67	0.25–1.77	0.478
*2DL3/2DL3*	30.6	39.3	30.0	36.0	0.78	0.43–1.43	0.453	1.15	0.50–2.63	0.505	0.76	0.28–2.10	0.804
***HLA-C*** ** alleles**													
*C1/C1*	23.6	25.0	30.0	18.7	1.35	0.68–2.66	0.475	1.45	0.56–3.75	0.443	1.87	0.66–5.29	0.241
*C1/C2*	43.1	39.3	45.0	54.0	0.64	0.37–1.13	0.152	0.55	0.24–1.26	0.216	0.70	0.27–1.78	0.483
*C2/C2*	33.3	35.7	25.0	27.3	1.33	0.72–2.44	0.431	1.48	0.63–3.46	0.370	0.89	0.30–2.59	1.000
***KIR-HLA*** ** combinations**													
*2DL1*+*C2*	76.4	75.0	70.0	81.3	0.74	0.38–1.47	0.476	0.69	0.27–1.78	0.443	0.54	0.19–1.52	0.241
*2DL2*+*C1*	50.0	39.3	50.0	45.3	1.21	0.69–2.12	0.566	0.78	0.34–1.78	0.679	1.21	0.47–3.07	0.812
*2DL3*+*C1*	47.2	53.6	50.0	60.7	0.58	0.33–1.02	**0.062**	0.75	0.33–1.68	0.532	0.65	0.25–1.65	0.468
*2DS1*+*C2*	6.9	3.6	5.0	6.7	1.04	0.34–3.18	0.999	0.52	0.06–4.22	1.000	0.74	0.09–6.08	1.000
*2DS2*+*C1*	45.8	35.7	45.0	41.3	1.20	0.68–2.12	0.564	0.79	0.34–1.82	0.677	1.16	0.45–2.97	0.812
*2DL1*+*C2C2*	33.3	35.7	25.0	27.3	1.33	0.72–2.44	0.431	1.46	0.62–3.43	0.370	0.89	0.30–2.59	1.000
*2DL2*+*C1C1*	19.4	21.4	20.0	12.7	1.66	0.78–3.55	0.226	1.88	0.68–5.23	0.238	1.72	0.52–5.70	0.482
*2DL3*+*C1C1*	16.7	21.4	20.0	14.0	1.23	0.57–2.66	0.687	1.68	0.61–4.62	0.387	1.54	0.47–5.04	0.502
*2DS1*+*C2C2*	4.2	3.6	0.0	1.3	3.22	0.53–19.70	0.332	2.74	0.24–31.30	0.403	0.00	0.00-NaN	1.000
*2DS2*+*C1C1*	18.1	21.4	15.0	12.0	1.62	0.74–3.51	0.223	2.00	0.72–5.59	0.224	1.29	0.34–4.86	0.718
*2DL2/2DL2*+*C1C1*	6.9	3.6	10.0	4.7	1.52	0.47–4.98	0.532	0.76	0.09–6.40	1.000	2.27	0.44–11.77	0.286
*2DL2/2DL2*+*C2C2*	2.8	0.0	5.0	6.7	0.40	0.09–1.88	0.345	0.00	0.00-NaN	0.366	0.74	0.09–6.08	1.000
*2DL2/2DL2*+*C1C2*	9.7	0.0	15.0	7.3	1.36	0.50–3.67	0.602	0.00	0.00-NaN	0.217	2.23	0.57–8.80	0.217
*2DL3/2DL3*+*C1C1*	4.2	3.6	10.0	6.0	0.68	0.18–2.60	0.755	0.58	0.07–4.77	1.000	1.74	0.35–8.70	0.621
*2DL3/2DL3*+*C2C2*	16.7	21.4	5.0	8.7	2.11	0.91–4.89	0.110	2.87	0.99–8.35	**0.086**	0.55	0.07–4.48	1.000
*2DL3/2DL3*+*C1C2*	9.7	14.3	15.0	21.3	0.40	0.17–0.95	**0.038**	0.61	0.20–1.90	0.608	0.65	0.18–2.36	0.769
*2DL2/2DL3*+*C1C1*	12.5	17.9	10.0	8.0	1.64	0.66–4.10	0.329	2.50	0.80–7.76	0.152	1.28	0.26–6.18	0.671
*2DL2/2DL3*+*C2C2*	12.5	14.3	10.0	11.3	1.12	0.47–2.65	0.825	1.30	0.40–4.21	0.749	0.87	0.19–4.08	1.000
*2DL2/2DL3*+*C1C2*	20.8	17.9	15.0	25.3	0.78	0.39–1.53	0.505	0.64	0.23–1.80	0.478	0.52	0.14–1.87	0.411

Bold *P* values indicate trends (0.05<*P*<0.1) or significant differences (*P*<0.05).

#### *KIR2DL2/KIR2DL2*

IP infants had a strong trend of lower representation of homozygous *KIR2DL2* compared to EU infants (*P* = 0.052; OR = 0.16), and this trend was maintained post MVL adjustment (Table 2: *P* = 0.058; OR = 0.13). Although this trend was not seen post NVP stratification (Table 3), within the mothers that received a single dose NVP, *KIR2DL2/KIR2DL2* showed an opposite deleterious trend of higher representation amongst INF infants compared to EU infants (*P* = 0.08; OR = 2.33), which again showed a trend when analysed in the context of low MVL (Table 2: *P* = 0.06; OR = 4.54).

#### *KIR2DL2/KIR2DL2+C1C2*

Although this combination did not show any significant association in the overall group comparisons, stratification according to NVP dose showed a trend (*P* = 0.06; OR = 4.46) of high representation in INF infants compared to EU infants in the group whose mothers received a single dose NVP (Table 3), however this did not withstand adjustment for MVL (Table 2).

#### *KIR2DL3+C1* and *KIR2DL3/KIR2DL3+C1C2*

The INF infants had a trend towards lower representation of the combination of *KIR2DL3* and C1 group HLA-C ligand compared to EU infants (*P* = 0.062; OR = 0.58). This was significant (*P* = 0.02; OR = 0.47) post MVL adjustment and also proved to be significant (*P* = 0.03; OR = 0.30) in the context of low MVL (Table 2). Upon stratification according to NVP dose, it was the INF infants born to mothers who received a single dose NVP that showed significant (*P* = 0.007; OR = 0.32) lower representation of this combination compared to the EU infants (Table 3), and again the significance was maintained post MVL adjustment (Table 2: *P* = 004; OR = 0.29) and in the context of low MVL (Table 2: *P* = 0.007; OR = 0.15) in this NVP-stratified subgroup.

The INF infant group also had significantly lower representation of the combination of homozygous *KIR2DL3* and heterozygous HLA-C allotype group i.e. *C1C2* (*P* = 0.038; OR = 0.40). This proved to be more significant post MVL adjustment (Table 2: *P* = 0.009; OR = 0.25) and was also significant upon NVP stratification in the group whose mothers received single dose NVP pre- (Table 3: *P* = 0.03; OR = 0.29) and post MVL adjustment (Table 2: *P* = 0.021; OR = 0.22).

#### *KIR2DL3/KIR2DL3+C2C2*

A weak trend of increased representation of homozygous *KIR2DL3* in the absence of its' HLA-C ligand (i.e. homozygous C2 group) in the IP infants compared to the EU infants was noted (*P* = 0.086; OR = 2.87). This proved to be significant (*P* = 0.008; OR = 5.57) post MVL adjustment and also significant (*P* = 0.05; OR = 5.18) in the context of low MVL (Table 2). Stratification according to NVP dose showed this genotypic combination to be significantly (*P* = 0.02; OR = 8.50) elevated in the IP infants vs. EU infants whose mothers did not receive NVP (Table 3), and this too withstood MVL adjustment (Table 2: *P* = 0.02; OR = 10.42) and proved to be significant (*P* = 0.02; OR = 48) in the context of low MVL (Table 2). In addition a trend (*P* = 0.07; OR = 5.10) of INF infants having higher representation of *KIR2DL3*/*KIR2DL3*+*C2C2* vs. EU infants post NVP stratification in the no maternal NVP subgroup was noted. When this subgroup was subjected to MVL adjustment, the trend was maintained (*P* = 0.072; OR = 6.16) and was significant (*P* = 0.03; OR = 23.8) in the context of low MVL (Table 2), however it also proved to be significantly elevated in INF infants vs. EU infants (*P* = 0.033; OR = 3.64) post MVL adjustment in the infant group whose mothers did receive a single dose NVP (Table 2).

#### *KIR2DL3/KIR2DL3+C1C2*

**Figure 2:**
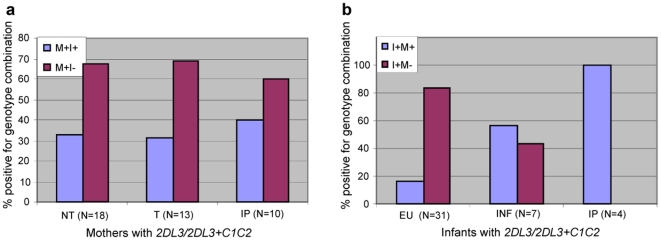
Maternal-infant concordance and discordance with respect to *KIR2DL3*/*KIR2DL3*+*C1C2*. **a**: Percentage of mother subgroups (all harbouring *KIR2DL3*/*KIR2DL3*+*C1C2*) that are concordant (M+I+) and discordant (M+I-) with their respective infants for the *KIR2DL3*/*KIR2DL3*+*C1C2* genotype. M: Mother; I: Infant; +: positive; -: negative; NT: non-transmitting mothers; T: total transmitting mothers; IP: intrapartum-transmitting mothers. **b**: Percentage of infant subgroups (all harbouring *KIR2DL3*/*KIR2DL3*+*C1C2*) that are concordant (I+M+) and discordant (I+M-) with their respective mothers for the *KIR2DL3*/*KIR2DL3*+*C1C2* genotype. EU: exposed-unifected infants; INF: total infected infants; IP: infants infected through the intrapartum route.

The genotype *KIR2DL3*/*KIR2DL3*+*C1C2* appears to have conflicting associations, i.e. associated with a higher chance of IP transmission in the mothers but an overall decreased susceptibility in the infants. We thus looked at the genotypes of matching mothers and infants to try and further elucidate the effect of *KIR2DL3/KIR2DL3+C1C2* in both transmission and susceptibility. These results are depicted in Figure 2 and show that in the mothers, irrespective of transmissibility (i.e. whether they are NT, TR or IP-transmitting mothers), approximately two thirds of all mothers harbouring the genotype are discordant with their infants for the genotype (i.e. mother positive for genotype and corresponding infant negative for genotype) compared to a third that are concordant (both mother and corresponding infant are positive for the genotype). In the infants however, the EU infants are distinctly different to the INF and IP infants with 84% of EU infants that harbour the genotype being discordant with their mothers and 16% being concordant. Among INF infants that harbour the genotype on the other hand, 57% are concordant with their mothers and 43% are discordant and among IP infants, all four IP infants positive for *KIR2DL3/KIR2DL3+C1C2*, are concordant with their mothers.

### The effect of HLA alone

Although the aim of this study was not to determine the role of HLA in maternal-infant HIV-1 transmission, since HLA has been shown in numerous previous studies to impact strongly on HIV-1 disease progression and to some extent on maternal-infant transmission, we conducted some preliminary analyses to determine if the effects we have detected for KIR-HLA are independent from the effects of HLA alone. Mother-infant HLA allele concordance, HLA homozygosity, individual HLA-B* and HLA-C* alleles and previously defined protective alleles [21] were investigated and results are shown in Table 6. No significant differences were detected when comparing mother-infant *HLA-B* and/or *HLA-C* allelic concordance, and, HLA homozygosity in the mothers did not show any significant associations with transmission (Table 6). Homozygosity in the HLA-B* locus showed a trend (*P* = 0.05; OR = 3.97) of higher representation in IP infants compared to EU infants (Table 6) and when comparing individual alleles, again only the HLA-B* locus appeared to be exerting an effect with B*08:01 showing a trend (*P* = 0.06; OR = 0.30) of lower representation in INF infants compared to EU infants, B*14:02 showing significantly (*P* = 0.03; OR = ∞) higher representation in TR mothers compared to NT mothers and B*42:01 showing a trend (*P* = 0.08; OR = 0.44) of lower representation in TR mothers compared to NT mothers (Table 6). No significant associations were seen with individual HLA-C* alleles. Looking at the combined effect of previously defined protective alleles (which again are all HLA-B* alleles), some protective effect seems to be exerted only in the context of IU transmission, where IU mothers showed a trend (*P* = 0.05; OR = 0.15) and significant (*P* = 0.04; OR = 0.15) lower representation of protective alleles compared to EU mothers when analysing the genotypic and allelic contributions, respectively (Table 6).

**Table 6:** Comparison of mother and infant groups with respect to HLA concordance, homozygosity, allele frequency and protective alleles.

HLA Comparison	Mothers^a^						Infants^b^					
	TR vs. NT		IP vs. NT		IU vs. NT		INF vs. EU		IP vs. EU		IU vs. EU	
	OR	*P*	OR	*P*	OR	*P*	OR	*P*	OR	*P*	OR	*P*
**Concordance** ***HLA-***												
B*	0.90	1.00	0.71	1.00	1.09	1.00	-	-	-	-	-	-
C*	0.63	0.32	0.60	0.58	0.59	0.74	-	-	-	-	-	-
B* and C*	0.59	0.43	0.77	1.00	0.00	0.37	-	-	-	-	-	-
B* or C*	0.76	0.56	0.57	0.58	1.26	0.75	-	-	-	-	-	-
**Homozygosity** ***HLA-***												
B*	0.82	1.00	1.68	0.43	0.74	1.00	1.83	0.33	3.97	**0.05**	0.00	1.00
C*	0.95	1.00	0.94	1.00	0.82	1.00	1.13	0.81	1.26	0.72	0.55	1.00
B* and C*	0.34	0.43	0.89	1.00	0.00	1.00	2.16	0.59	2.72	0.41	0.00	1.00
B* or C*	1.07	0.84	1.34	0.57	1.02	1.00	1.27	0.66	2.12	0.21	0.41	0.70
**Alleles** c ***HLA-***												
B*08:01	0.74	0.55	ND	ND	ND	ND	0.30	**0.06**	ND	ND	ND	ND
B*14:02	∞	**0.03**	ND	ND	ND	ND	1.41	0.66	ND	ND	ND	ND
B*42:01	0.44	**0.08**	ND	ND	ND	ND	0.56	0.30	ND	ND	ND	ND
**Protective alleles** d												
Genotypic	0.57	0.13	0.59	0.36	0.15	**0.05**	0.60	0.23	0.49	0.24	0.73	0.78
Allelic	0.54	0.07	0.56	0.30	0.15	**0.04**	0.67	0.27	0.65	0.51	0.74	0.80

a : TR (Transmitting mothers; N = 68–74); NT (Non-transmitting mothers; N = 143–150); IP (Intrapartum-transmitting mothers; N = 28–29); IU (Intrauterine-transmitting mothers; N = 19–21).

b : INF (Total infected infants; N = 70–72); EU (Exposed-uninfected infants; N = 149); IP (Intrapartum-infected infants; N = 28); IU (Intrauterine-infected infants; N = 20).

c : Results are shown only for alleles where trends or significant differences were noted in either group (i.e. mothers and/or infants).

d : Protective alleles are HLA-B*57:02, HLA-B*57:03, HLA-B*58:01 and HLA-B*81:01. In the genotypic comparisons the individual was scored once for possession of at least one protective allele.

(i.e. if homozygous for protective allele was only scored once) and denominator was the number of individuals. In the allelic comparisons the number of alleles were scored and the denominator would be the total number of alleles (i.e. accounts for allelic dose of a protective allele).

-: Not applicable since concordance looks at mother-infant pairs.

ND: Not determined.

Bold *P* values indicate trends (0.05<*P*<0.1) or significant differences (*P*<0.05).

## Discussion

This study was conducted in order to investigate the role of KIR-HLA in maternal-to-infant HIV-1 transmission. This model of transmission allows one to determine influence at both the level of infant susceptibility as well as maternal transmissibility. By comparing mothers who transmitted to the mothers who did not transmit HIV-1, a possible role of *KIR2DL2* was indicated. The two KIR genotypes Bx32 and Bx20 showed opposing effects on transmission with Bx32 having significantly higher representation in TR mothers compared to NT mothers, which appears to be largely contributed by the IP-transmitting mothers (Figure 1b) and although the TR vs. NT association was weakened with MVL adjustment, the IP vs. NT association was more significant post-adjustment (Table 2). Bx20 on the other hand, had significantly higher representation in NT mothers compared to TR mothers (Figure 1b) and this association was also weakened with MVL adjustment but showed a similar trend (Table 2). The only apparent difference between these two genotypes is the absence of *KIR2DL2* in Bx32 (Figure 1C); however they may very well vary in the composition of the two parental haplotypes making up the combined genotype. In addition, NT mothers had higher representation of *KIR2DL2* compared to TR mothers which appears to be independent of whether transmission was IP or IU (Table S1). The heterozygous allelic combination of *KIR2DL2*/*KIR2DL3* was also significantly elevated in NT mothers compared to TR mothers and most pronounced when restricted to IP-transmitting mothers (Table 4), with both these associations withstanding MVL adjustments (Table 2). Combination of *KIR2DL2* homozygosity (*KIR2DL2*/*KIR2DL2*) and homozygosity for its HLA-C ligand (*C1C1*) however was significantly higher in TR mothers compared to NT mothers (Table 4), although this significance was lost upon MVL adjustment (Table 2) suggesting that viral load may be skewing the effect seen for this KIR-HLA combination. The data indicate a possible role for *KIR2DL2*, when found in heterozygous combination with *KIR2DL3*, with those mothers being less likely to transmit through the intrapartum route. The only reported study [16] involving a role for *KIR2DL2* in HIV-1 transmission found significantly higher frequencies of *KIR2DL2* in exposed seronegative African sex workers compared to seropositive sex workers (*P* = 0.004) as well as significantly more *KIR2DL2*/*KIR2DL3* heterozygosity among exposed seronegative sex workers compared to seropositive sex workers (*P* = 0.028; OR = 4.88). These associations are thus similar to the associations seen in our study, despite the different modes of transmission.

The other *KIR* gene that appears to be playing a role in maternal transmission of HIV-1 is *KIR2DL3*, where homozygosity for this gene was significantly more highly represented in IP-transmitting mothers compared to NT mothers and *KIR2DL3* homozygosity in the presence of *C1C2* heterozygosity was significantly elevated in IP-transmitting mothers compared to NT mothers (Table 4). These two relationships remained significant post MVL adjustment and in addition proved to be significant in the context of low MVL (Table 2), suggesting that high viral load may be masking the effect of the genes. Interestingly, *KIR2DL3* and HLA-C1 have also been shown to play a role in the resolution of hepatitis C virus (HCV) in the context of low infectious doses of HCV but not in individuals with high-dose exposure [22]. Jennes *et al*. [16] also report seropositive sex workers to show markedly higher proportion of *KIR2DL3* homozygosity (*P* = 0.004; OR = 0.11) and *KIR2DL3* homozygosity in the presence of *C1C2* heterozygosity (*P* = 0.015; OR = 0.13) when compared to exposed seronegative sex workers. Although the current study and the Jennes *et al*. [16] study seem to correlate with regard to the *KIR* genes and KIR-HLA combinations that show significant associations, the actual mechanisms that may be at play remain elusive. Jennes *et al*. [16] also report and stress the association of certain inhibitory *KIR* genes in the absence of their cognate *HLA* genes with exposed seronegative status, namely *KIR2DL2*/*KIR2DL3* in the absence of *HLA-C1* and *KIR3DL1* homozygosity in the absence of *HLA-Bw4*, and thus propose that the absence of HLA ligands for inhibitory KIR may lower the threshold for activation of NK cells via activating KIR, thereby resulting in early elimination of HIV-1 infected cells by NK cytotoxic activity. These associations however were not seen in our study.

Most of the significant associations seen in maternal HIV-1 transmission were pertaining to the intraparum route, however IU-transmitting mothers overall had a higher representation of the AA haplotype group and lower representation of the Bx haplotype group (more activating genes) when compared to the other maternal groups (Fig. 1a), suggesting that a more inhibitory NK cell effector function increases likelihood of intrauterine transmission. IU-transmitting mothers had a trend of lower total *KIR* gene number and significantly lower number of inhibitory *KIR* genes compared to NT mothers (Table 1), in agreement with the high AA haplotype group representation. Overall, fewer genes could imply overall less immune activation which may be more conducive to intrauterine transmission and it could very well be the balance or ratio of select inhibitory *KIR* to activating *KIR* genes which is playing a role rather than a predominance of one or the other. The significant associations of lower total *KIR* gene number and inhibitory *KIR* genes in the IU-transmitting mothers vs. NT mothers were maintained post-MVL adjustment (Table 2).

A comparison of infants that become infected with HIV-1 to those that are exposed but remain uninfected is an ideal model for investigating the contribution of host genes to increased HIV-1 susceptibility or protection. In this study no significant differences were observed in the individual *KIR* gene comparisons, *KIR* gene number and ratios or *KIR* genotype, however the combination of *KIR3DL1* and its HLA-B ligand, *Bw480Ile*, showed a trend of higher representation in EU infants compared to IP infants (Table S4), this trend however was lost upon MVL adjustment (Table 2). Previous studies have implicated this KIR-HLA combination in both HIV-1 disease progression [17], [19] and risk of HIV-1 infection [13] and interestingly it is the combination of high expressing *KIR3DL1* alleles as well as the cell-retained receptor expressed by the KIR3DL1*004 allele, in combination with *Bw480Ile* (often the HLA-B*57 allele) that confer a strong protective effect. We failed to see any significant associations with the KIR3DL1*004 allele+*Bw480Ile*, however it may be possible that the trend that was observed could be intensified by comparing *KIR3DL1* high expressing alleles in combination with *Bw480Ile* and by selectively looking at the HLA-B*57 expressing individuals amongst the *Bw480Ile* group.

Additional trends and significant associations seen within the infant group again involved *KIR2DL2* and *KIR2DL3*. Homozygosity for *KIR2DL2* showed a strong trend for underrepresentation in IP infants compared to EU infants (Table 5) which was maintained upon MVL adjustment (Table 2), however no significant associations were observed with the combination of this *KIR* gene and its cognate HLA-C1 ligand. On the other hand *KIR2DL3* in combination with its HLA-C1 ligand showed a trend of low representation amongst INF infants compared to EU infants and a combination of homozygosity for *KIR2DL3* and heterozygosity for the HLA-C ligand (*C1C2*) showed significant low representation among INF infants compared to EU infants (Table 5). These associations were strengthened upon MVL adjustment and *KIR2DL3*+*C1* also proved to be significantly elevated in EU infants compared to INF infants in the low MVL group (Table 2), again suggesting that the impact contributed by genotype is seen under conditions of low viral load and that high viral load appears to override the effect of host genotype. This association is opposite to the effect seen of this same combination in the mothers and by Jennes *et al*. [16]. To investigate this further we looked at the level of concordance between mothers and infants harbouring this genotype, and the results (Figure 2) point to this genotype being protective in the infant only when there is discordance i.e. the mother lacking the genotype, and possibly that the protection is in the context of IP transmission as the three INF infants (43%) that were discordant for the genotype were all IU-infected infants.

The trend seen with IP infants having higher representation of homozygosity for *KIR2DL3* and *C2* compared to EU infants was substantially more significant post MVL adjustment and also appears to be apparent under conditions of low maternal viral load (Table 2), again highlighting a role for *KIR2DL3*, in the absence of its ligand, with increased infant IP-susceptibility to HIV-1.

No significant associations were seen in either the mothers or the infant group with regard to *KIR3DS1* alone or in combination with its Bw4 ligand, a *KIR* gene and HLA-KIR combination that has been studied and reported on widely in relation to HIV-1 disease progression in the literature [12], [14], [15], [18], [23]. *KIR3DS1* however is found at a very low frequency in our study population (<10%; Tables S1 and S2), and may thus not be contributing a role that is detectable at a population level.

Antiretroviral drugs play a role in reducing maternal-to-infant HIV-1 transmission, and given that a previous study has seen the protective effect of high *CCL3L* gene copies obscured by the presence of maternal single dose NVP [24], we analysed our study population according to maternal NVP dose. Absence of maternal NVP failed to reveal strong associations that were previously not there and did not appear to significantly strengthen any associations already seen in both the mother and infant groups. Some previously significant differences that were lost are most likely due to the smaller numbers being compared. Many of the trends that were strengthened or lost in both the presence and absence of maternal NVP, were often as a result of the skewing of the data due to the no maternal NVP mother and infant groups having a higher proportion of IP mothers and infants respectively and the maternal NVP mother and infant groups having higher proportions of IU mothers and infants respectively (see footnote Table 7). This is not unexpected since administration of NVP at labour serves to reduce IP infection and thus one would expect a skewing towards fewer IP transmissions and infections in the group of mothers that received NVP and visa versa. Some associations however seemed not to be affected by the skewed data. The Bx20 genotype which was no longer significantly underrepresented in TR mothers vs. NT mothers in the absence of maternal NVP, maintained its significance in the presence of maternal NVP. Also, in the presence of maternal NVP, the trend of low representation of *KIR2DL3* and its *C1* ligand in INF infants vs. EU infants, was shown to be highly significant (*P* = 0.007). This association was strengthened post MVL adjustment and highly significant in the context of low MVL (Table 2). *KIR2DL3* homozygosity and heterozygosity for the HLA-C ligand (*C1C2*), maintained significant low representation in INF vs. EU infants, only in the presence of maternal NVP, and was maintained post MVL adjustment again.

**Table 7:** Distribution of study participants (total and maternal nevirapine-stratified groups) according to cohorts previously described and maternal viral load (VL) and CD4 counts.

	Cohorts*				Total	VL (copies/ml)log_10_	CD4 (cells/µl)
	1	2	3	4			
	N =				N =	Median (range)	Median (range)
Total groups							
NT mothers	39	56	14	41	150	3.97 (1.70–5.88)N = 136	450 (16–1655)N = 137
TR mothers	22	25	7	20	74	4.79 (2.60–5.87)N = 66	375 (25–1026)N = 65
EU infants	41	51	17	41	150	-	-
INF infants	22	24	6	20	72	-	-
Maternal nevirapine (NVP) stratified groups							
Maternal NVP							
NT mothers	9	35	0	38	82	3.89 (2.60–5.88)N = 82	474 (16–1479)N = 72
TR mothers^a^	5	19	0	18	42	4.49 (2.60–5.87)N = 41	390 (25–1011)N = 36
EU infants	9	34	0	38	81	-	-
INF infants^c^	5	18	0	18	41	-	-
No maternal NVP							
NT mothers	30	21	1	0	52	3.92 (1.70–5.69)N = 51	565 (125–1655)N = 49
TR mothers^b^	17	6	1	0	24	4.73 (2.92–5.79)N = 23	444 (127–1026)N = 21
EU infants	32	17	4	0	53	-	-
INF infants^d^	17	6	1	0	24	-	-

-: Not applicable (EU infants) and not determined (INF infants).

*: Described in Kuhn *et al*. (2007).

a :10 IPs (43%) + 13 IUs (56%) = 23 (known mode of transmission); 19 = unknown mode of transmission.

b :16 IPs (69.6%) + 7 IUs (30.4%) = 23 (known mode of transmission); 1 = unknown mode of transmission.

c :10 IPs (45.5%) + 12 IUs (54.6%) = 22 (known infection route); 19 = unknown infection route.

d :16 IPs (69.6%) + 7 IUs (30.4%) = 23 (known infection route); 1 = unknown infection route.

Since it is in the presence of maternal NVP that most of the associations remained significant or were strengthened, this raises a number of postulations. Firstly, the possibility that NVP is lowering MVL, however with administration of NVP at labour, it is unlikely that the peripheral viral loads would be reduced to the extent that the infant is exposed to fewer viruses. In support, comparison of MVL between mothers that received NVP to those that did not (since blood was drawn at labour) showed that there was no significant difference between the two groups (data not shown). It is generally considered that it is the exposure of the infant to maternal NVP, which crosses the placenta, that helps prevent the establishment of HIV-1 infection in the infant. Thus, low MVL and exposure to maternal NVP result in a similar outcome, namely a reduced risk of infant to infection and possibly the ability for select genotypes to reveal their effect. Lastly, it is possible that NVP is having a modulatory effect on certain immune pathways that are somehow enhancing the effects of select genotypes. Immunomodulatory effects of NVP in infants born to HIV-1 seropositive mothers have previously been reported [25]. This study serves to highlight once again that the study of genes that impact on maternal-to-infant HIV-1 transmission cannot be done so in the absence of accounting for the presence of the antiretroviral drugs.

A preliminary investigation into the influence of HLA alone on mother-to-infant HIV-1 transmission in this cohort and whether this may be influencing the results of this study, revealed that only the HLA-B* locus seemed to be exerting some effect (Table 6). In a previous study where maternal HLA homozygosity and HLA concordance was linked to increased risk of vertical HIV-1 transmission [26], this was more apparent with IU and breast milk transmission, and since breast milk transmission was not a factor in this study and only 21 out of 74 mothers in this study were confirmed to transmit via the IU route (vs. 29 IP transmissions), the remaining 24 unknown transmission routes may be predominantly of the IP route. Furthermore, the Mackelprang *et al*. study [26] was also carried out on a cohort receiving antenatal zidovudine which could be influencing transmission in its own right. Nevertheless, the absence of a contribution of HLA-C* on mother-to-infant transmission in our study further strengthens the significant associations seen with *KIR* and HLA-C*.

Due to the hypothesis-generating nature of this study, rather than a wide-ranging screening for genes and associations, correction for multiple testing was not applied. This appears to be in agreement with other studies that have investigated the role of KIR-HLA in viral transmission and disease progression [22], [24]. Adjustment for multiple comparisons corrects for type 1 errors but increases the risk of type 2 errors. Given the complexity and multifactorial nature of maternal-infant HIV-1 transmission, we considered it more important to identify potential factors that may play a role in this route of infection rather than simply dismissing these leads as due to chance variations brought about by multiple comparisons. Nevertheless, it remains important that the consistency of these associations in other cohorts be investigated.

There are a number of limitations of the current study and areas that we feel require further investigation to elucidate the exact role of KIR-HLA on mother-to-infant HIV-1 transmission. Firstly, although we looked at mother-infant genotype concordance with respect to *KIR2DL3/KIR2DL3*+*C1C2*, this was only conducted in order to try and understand the apparent contradictory effect of this particular genotype. However, this has served to highlight the importance of looking at mother-infant genotypic combinations with respect to the remaining KIR-HLA combinations. Furthermore, allorecognition of infected maternal cells in the infant may well contribute to protection from infection, thus analysis of the infant KIR vs. the corresponding mother's HLA can serve to determine if allorecognition is a contributing factor. It is important to note that this study simply looked at *KIR* gene content and does not account for allelic variation or the fact that having a particular *KIR* gene does not necessarily equate into expression of that gene and the presence of that receptor on the cell surface. In addition, HLA-G serves as a ligand for KIR2DL4 and is thought to play a role in IU transmission, however, given that most individuals have the *KIR2DL4* gene, this too would need to be addressed at the allelic level. HLA-G polymorphisms have also been described that may very well affect KIR-HLA interactions and impact on vertical transmission.

In conclusion, this study is the first to investigate the role of KIR and KIR-HLA in HIV-1 maternal-to-infant transmission, and suggests a role for *KIR2DL2*, *KIR2DL3* with their cognate *HLA-C* ligands in HIV-1 transmission and susceptibility in the context of low maternal viral load and/or maternal nevirapine.

## Materials and Methods

### Study participants

A total of 224 mothers and 222 infants, recruited as part of 4 mother-to-infant HIV-1 transmission cohorts in Johannesburg, South Africa, were used in this study. A detailed description of the 4 cohorts is given by Kuhn *et al*. [24]. All available transmitting pairs (infant infected) and a random sample of two non-transmitting pairs (mother infected but infant uninfected) per case were selected from each cohort using a case-cohort design. Since complete samples were not available for every pair, of the 446 individuals, 145 were matching mother-infant pairs where the infants were HIV-1 exposed but remained uninfected (EU), and there were 5 unmatched mothers and 5 unmatched infants thereby totalling 150 EU infants and 150 non-transmitting (NT) mothers. There were 72 matching mother-infant pairs where the infant was determined HIV-1 infected and two unmatched mothers thereby totalling 72 infected (INF) infants and 74 transmitting (TR) mothers. The infant's infection status was determined by a HIV-1 DNA PCR test (Roche Amplicor version 1.5). Of the 72 INF infants, 20 were infected in utero (IU; PCR positive at birth), 28 were infected intrapartum (IP; PCR negative at birth, positive at 6 weeks postpartum) and the remaining 24 were found to be infected at 6 weeks but had no birth sample available for determining the timing of transmission. In the case of TR mothers, 23% in our study reported any breastfeeding vs. 16% of NT mothers (not significant). The average duration of breastfeeding for those who initiated any breastfeeding was 14 days. By extrapolation the effect of breastfeeding would be negligible in this cohort. Moreover as breastfeeding and IP transmission are thought to occur across mucosal surfaces vs. IU which seems a more direct into the system exposure, thus, combining the majority IP with a tiny proportion of breastfeeding would not confound the genetic outcomes with transmission likelihood.

Since this study aims at investigating the role of select genes on mother-to-infant HIV-1 transmission, one cannot exclude the role played by the various antiretroviral drugs administered in the 4 cohorts studied here. In addition to looking at the overall groups, we stratified the study participants according to whether the mothers were administered no nevirapine (NVP) or a single-dose NVP during labour. All infants received a single dose of NVP. Mothers and infants that were administered other antiretroviral drugs were excluded from this part of the analysis.

Since not all the individuals described by Kuhn *et al*. [24] were used in this study, Table 7 shows the distribution of the study participants across the 4 cohorts. The median maternal viral loads (copies/ml) and median CD4 counts (cells/µl) for the broad groups are also shown in Table 7. The viral load determinations were done on maternal delivery samples using the Roche Amplicor RNA Monitor assay version 1.5 (Roche Diagnostic Systems, Inc., Branchburg, NJ) and the CD4 T-cell counts were determined using the commercially available FACSCount System from Becton Dickinson (San Jose, CA).

This study was approved by the University of the Witwatersrand Committee for Research on Human Subjects and the Institutional Review Board of Columbia University and signed informed consent was obtained from all mothers who participated in the study.

### Genomic DNA extraction

Genomic DNA used for both the *KIR* as well as the *HLA* genotyping was extracted from 5 ml whole blood using the QIAamp DNA Blood Mini Kit (Qiagen, Dusseldorf, Germany) according to the manufacturer's instructions.

### KIR genotyping

*KIR* genotying was performed using sequence-specific primer (SSP) PCR (*Olerup* SSP KIR Genotyping kit; *Olerup* SSP AB, Sweden). KIR locus typing was carried out to detect the presence or absence of the following 14 *KIR* genes: *KIR2DL1*, *KIR2DL2*, *KIR2DL3*, *KIR2DL4*, *KIR2DL5*, *KIR2DS1*, *KIR2DS2*, *KIR2DS3*, *KIR2DS4*, *KIR2DS5*, *KIR3DL1*, *KIR3DL2*, *KIR3DL3*, *KIR3DS1*, and 2 pseudogenes: *KIR2DP1* and *KIR3DP1*. The kit also allowed allelic resolution of *KIR3DL1* for detection of KIR3DL1*004.

Group B haplotypes were defined by the presence of one or more of the following genes: *KIR2DL5*, *KIR2DS1*, *KIR2DS2*, *KIR2DS3, KIR2DS5,* and *KIR3DS1*. Group A haplotypes were defined by the absence of all group B genes and the presence of *KIR2DL1*, *KIR2DL3*, *KIR2DL4*, *KIR2DS4*, *KIR2DP1*, *KIR3DL1*, *KIR3DL2*, *KIR3DL3* and *KIR3DP1* (14^th^ International HLA and Immunogenetics Workshop, 2005). The group B haplotypes were collectively termed Bx, since they represent a mixture of AB and BB haplotypes. Furthermore, *KIR* genotype profiles were assigned to the AA and Bx haplotype groups using the New Allele Frequency Database: http://www.allelefrequencies.net [20].

### HLA genotyping

*HLA-B* and *HLA-C* high resolution genotying was performed using a sequence-based typing (SBT) strategy using the protocol described for heterozygous amplification of exon 2, intron 2, and exon 3 of the HLA loci [27]. Nucleotide sequencing was performed on an ABI 3730 Genetic Analyzer using Big Dye Terminator v1.1 chemistry (Applied Biosystems, Foster City, CA). Allele assignment was performed using SeqScape v2.5 software (Applied Biosystems) and a library compiled from the 2.17.0 release of the IMGT/HLA Database.

*HLA-B* and *HLA-C* alleles were classified into their respective allotype groups based on the following: HLA-B molecules express one of two mutually exclusive epitopes, Bw4 and Bw6 determined by 5 variable amino acids spanning positions 77-83 [28]. Furthermore the Bw4 epitopes can be divided into two groups, namely Bw4-80Ile and Bw4-80Thr depending on the presence of isoleucine or threonine at position 80 respectively [29]. HLA-C alleles can be divided into two groups, C1 and C2 groups based on the position of an asparagine or lysine residue at position 80 respectively [30].

### Test for Hardy-Weinberg (HLA)

Overall deviations from Hardy-Weinberg proportions for the *HLA-B* and *HLA-C* alleles were tested using the conventional Monte Carlo exact test of Guo and Thompson (1992) [31] implemented through the computer program TFPGA (Tools for Population Genetic Analyses version 1.3; 1997: author Mark. P. Miller). The mother and infant groups were tested separately.

### KIR-HLA Analysis

Frequencies of KIR A and B haplotypes and genotypes, HLA-B and HLA-C allotype groups, individual *KIR* genes as well as combinations of select *KIR* genes and their known ligands were determined and compared between the appropriate groups. *KIR2DL2* and *KIR2DL3* encode receptors that bind to C1 group HLA-C molecules. KIR2DL1 binds C2 group HLA-C molecules and KIR3DL1 binds to Bw4 HLA-B molecules. Several studies [29], [32], [33] have shown that Bw4-80Ile molecules serve as better ligands for KIR3DL1 than Bw4-80Thr molecules. KIR2DS1 and KIR2DS2 bind to C2 group and C1 group HLA-C allotypes respectively. Although the ligand for KIR3DS1 is not definitively known, its high (>95%) amino acid sequence similarity in its extracellular domain with KIR3DL1, and data showing that the combination of KIR3DS1-Bw4-80Ile has been associated with slow progression to AIDS [18], suggest Bw4 molecules as putative ligands of this receptor.

Fisher's exact tests were used to calculate statistical significances and exact 95% confidence intervals (CI) of odds ratios (OR) of genotype frequency differences (*SISA*: Simple Interactive Statistical Analysis; [34]). Two-sided tests were used and the level of statistical significance for analyses was set at *P*<0.05. No adjustment was made for multiple comparisons.

In addition, the contribution of *KIR* gene number as well as the balance between activating and inhibitory *KIR* (ratio of activating:inhibitory *KIR* gene number) was compared between groups by using the non-parametric Mann-Whitney *U* test (SPSS software version 15.0; SPSS Inc.).

Multivariable logistic regression was used to adjust for maternal viral load (MVL) and stratified analyses of the associations between genotypes and transmission was conducted among women with high and low MVL defined as being above or below the median viral load for the study population overall.

### The effect of HLA alone

To determine if *HLA* alone was impacting on maternal-infant HIV-1 transmission and thereby confounding the results seen with the *KIR-HLA* analysis, we investigated the effect of *HLA* by looking at the influence of a number or factors, namely:

#### Maternal-infant HLA concordance

Concordance was scored as the number of shared *HLA-B* and *HLA-C* alleles, *HLA-A* data was not available for this cohort and thus could not be looked at. Mothers and infants share at least one allele at each locus. We analysed the effect of concordance (two shared alleles) in each locus separately as well as the effect of combined concordance (i.e. concordant at both loci) and the effect of concordance in either locus (i.e. *HLA-B* or *HLA-C* concordance).

#### HLA homozygosity

The effect of maternal and infant homozygosity at the *HLA-B* and *HLA-C* loci was looked at independently and in addition, the effect of combined homozygosity (i.e. both loci homozygous) and homozygosity at one or the other locus (i.e. *HLA-C* or *HLA-B*) was investigated.

#### HLA alleles

The effect of individual *HLA-B* and *HLA-C* alleles on both transmission and infant susceptibility were investigated by comparing the frequencies of all alleles present in the respective groups. These comparisons were only carried out on the broad groups (i.e. TR vs. NT mothers and INF vs. EU infants).

#### HLA protective alleles

The following alleles have been previously shown to be associated with low viral loads and high CD4 counts in a cohort of HIV-I-infected adults in South Africa: HLA-B*57:02, HLA-B*57:03, HLA-B*58:01 and HLA-B*81:01 [21]. We looked at the combined effect of these alleles on both transmission and susceptibility by comparing the combined frequencies of these alleles between respective groups. Both genotypic and allelic frequencies were compared.

Fisher's exact test was used to calculate statistical significances between the groups above. Correction for MVL and stratification according to maternal NVP dose were not carried out on the HLA comparisons and no adjustment was made for multiple comparisons.

## Supporting Information

Table S1 Comparison of frequencies of *KIR* genes between HIV-1-transmitting (TR) mothers, intrapartum (IP)-HIV-1-transmitting mothers, intrauterine (IU)-HIV-1-transmitting mothers and non-transmitting (NT) mothers.
(DOC)

Table S2 Comparison of frequencies of *KIR* genes between HIV-1-infected (INF) infants, intrapartum (IP)-HIV-1-infected infants, intrauterine (IU)-HIV-1-infected infants and exposed-uninfected (EU) infants.
(DOC)

Table S3 Comparison of frequencies of *KIR3DL1*, *KIR3DS1* and *HLA-Bw* allotypes as well as *KIR-HLA-Bw* combinations between HIV-1-transmitting (TR) mothers, intrapartum (IP)-HIV-1-transmitting mothers, intrauterine (IU)-HIV-1-transmitting mothers and non-transmitting (NT) mothers.
(DOC)

Table S4 Comparison of frequencies of KIR3DL1, KIR3DS1 and HLA-Bw allotypes as well as KIR-HLA-Bw combinations between HIV-1-infected (INF) infants, intrapartum (IP)-HIV-1-infected infants, intrauterine (IU)-HIV-1-infected infants and exposed uninfected (EU) infants.
(DOC)
