# Supplementary material for: KIR-HLA and Maternal-Infant HIV-1 Transmission in Sub-Saharan Africa
Source: PLoS One. 2011 Feb 4;6(2):e16541. doi: 10.1371/journal.pone.0016541 (PMC3035631; doi:10.1371/journal.pone.0016541)
Supplement: Table S1 — Comparison of frequencies of KIR genes between HIV-1-transmitting (TR) mothers, intrapartum (IP)-HIV-1-transmitting mothers, intrauterine (IU)-HIV-1-transmitting mothers and non-transmitting (NT) mothers. (DOC) [file pone.16541.s001.doc]

Table S1. Comparison of frequencies of *KIR* genes between HIV-1-transmitting (TR) mothers, intrapartum (IP)-HIV-1-transmitting mothers, intrauterine (IU)-HIV-1-transmitting mothers and non-transmitting (NT) mothers

| ***KIR* Genes** | **TR**  **mothers**  **(N=74)** | **IP mothers (N=29)** | **IU**  **mothers**  **(N=21)** | **NT**  **mothers (N=150)** |  | **TR mothers vs NT mothers** | | |  | **IP mothers vs NT mothers** | | |  | **IU mothers vs NT mothers** | | |
| --- | --- | --- | --- | --- | --- | --- | --- | --- | --- | --- | --- | --- | --- | --- | --- | --- |
|  | **% representation** | | | |  | **OR** | **95% CI** | ***P*** |  | **OR** | **95% CI** | ***P*** |  | **OR** | **95% CI** | ***P*** |
| *KIR2DL1* | 97.3 | 100.0 | 95.2 | 99.3 |  | 0.24 | 0.02-2.71 | 0.254 |  |  | NaN- | 1.000 |  | 0.13 | 0.01-2.23 | 0.231 |
| *KIR2DL2* | 59.5 | 48.3 | 52.4 | 68.7 |  | 0.67 | 0.38-1.19 | 0.181 |  | 0.43 | 0.19-0.95 | **0.053** |  | 0.50 | 0.20-1.26 | 0.146 |
| *KIR2DL3* | 71.6 | 72.4 | 76.2 | 78.0 |  | 0.71 | 0.38-1.34 | 0.321 |  | 0.74 | 0.30-1.82 | 0.481 |  | 0.90 | 0.31-2.65 | 0.786 |
| *KIR2DL4* | 98.7 | 100.0 | 95.2 | 100.0 |  | 0.00 | 0.00-NaN | 0.330 |  | - | - | - |  | 0.00 | 0.00-NaN | 0.123 |
| *KIR2DL5* | 64.9 | 69.0 | 47.6 | 65.3 |  | 0.98 | 0.55-1.76 | 1.000 |  | 1.18 | 0.50-2.77 | 0.832 |  | 0.48 | 0.19-1.21 | 0.148 |
| *KIR2DS1* | 16.2 | 20.7 | 9.5 | 10.7 |  | 1.62 | 0.72-3.63 | 0.283 |  | 2.18 | 0.77-6.16 | 0.134 |  | 0.88 | 0.19-4.14 | 1.000 |
| *KIR2DS2* | 60.8 | 51.7 | 52.4 | 58.0 |  | 1.12 | 0.64-1.98 | 0.773 |  | 0.78 | 0.35-1.72 | 0.546 |  | 0.80 | 0.32-1.99 | 0.645 |
| *KIR2DS3* | 27.0 | 27.6 | 19.1 | 27.3 |  | 0.98 | 0.53-1.84 | 1.000 |  | 1.01 | 0.42-2.47 | 1.000 |  | 0.63 | 0.20-1.97 | 0.598 |
| *KIR2DS4* | 98.7 | 100.0 | 95.2 | 98.7 |  | 0.99 | 0.09-11.06 | 1.000 |  |  | NaN- | 1.000 |  | 0.27 | 0.02-3.12 | 0.327 |
| *KIR2DS5* | 52.4 | 55.2 | 33.3 | 48.7 |  | 1.11 | 0.64-1.94 | 0.777 |  | 1.30 | 0.58-2.89 | 0.549 |  | 0.53 | 0.20-1.38 | 0.244 |
| *KIR2DP1* | 97.3 | 100.0 | 95.2 | 99.3 |  | 0.24 | 0.02-2.71 | 0.254 |  |  | NaN- | 1.000 |  | 0.13 | 0.01-2.23 | 0.231 |
| *KIR3DL1* | 98.7 | 100.0 | 95.2 | 99.3 |  | 0.49 | 0.03-7.94 | 0.553 |  |  | NaN- | 1.000 |  | 0.13 | 0.01-2.23 | 0.231 |
| *KIR3DL2* | 100.0 | 100.0 | 100.0 | 100.0 |  | - | - | - |  | - | - | - |  | - | - | - |
| *KIR3DL3* | 100.0 | 100.0 | 100.0 | 100.0 |  | - | - | - |  | - | - | - |  | - | - | - |
| *KIR3DS1* | 4.1 | 6.9 | 0.0 | 5.3 |  | 0.75 | 0.19-2.91 | 1.000 |  | 1.31 | 0.26-6.53 | 0.666 |  | 0.00 | 0.00-NaN | 0.598 |
| *KIR3DP1* | 98.6 | 100.0 | 95.2 | 100.0 |  | 0.00 | 0.00-NaN | 0.330 |  | - | - | - |  | 0.00 | 0.00-NaN | 0.123 |

Bold *P* values indicate trends (0.05<*P*<0.1) or significant differences (*P*<0.05)
