# Supplementary material for: KIR-HLA and Maternal-Infant HIV-1 Transmission in Sub-Saharan Africa
Source: PLoS One. 2011 Feb 4;6(2):e16541. doi: 10.1371/journal.pone.0016541 (PMC3035631; doi:10.1371/journal.pone.0016541)
Supplement: Table S2 — Comparison of frequencies of KIR genes between HIV-1-infected (INF) infants, intrapartum (IP)-HIV-1-infected infants, intrauterine (IU)-HIV-1-infected infants and exposed-uninfected (EU) infants. (DOC) [file pone.16541.s002.doc]

Table S2. Comparison of frequencies of *KIR* genes between HIV-1-infected (INF) infants, intrapartum (IP)-HIV-1-infected infants, intrauterine (IU)-HIV-1-infected infants and exposed-uninfected (EU) infants

| ***KIR* Genes** | **INF infants***  **(N=72)** | **IP infants**  **(N=28)** | **IU infants**  **(N=20)** | **EU infants**  **(N=150)** |  | **INF infants vs EU infants** | | |  | **IP infants vs EU infants** | | |  | **IU infants vs EU infants** | | |
| --- | --- | --- | --- | --- | --- | --- | --- | --- | --- | --- | --- | --- | --- | --- | --- | --- |
|  | **% representation** | | | |  | **OR** | **95% CI** | ***P*** |  | **OR** | **95% CI** | ***P*** |  | **OR** | **95% CI** | ***P*** |
| *KIR2DL1* | 98.6 | 100.0 | 95.0 | 99.3 |  | 0.48 | 0.03-7.73 | 0.544 |  |  | NaN- | 1.000 |  | 0.13 | 0.01-2.12 | 0.222 |
| *KIR2DL2* | 65.3 | 53.6 | 65.0 | 63.3 |  | 1.09 | 0.60-1.96 | 0.881 |  | 0.67 | 0.30-1.51 | 0.398 |  | 1.08 | 0.40-2.86 | 1.000 |
| *KIR2DL3* | 76.4 | 89.3 | 65.0 | 80.7 |  | 0.78 | 0.39-1.53 | 0.482 |  | 2.00 | 0.56-7.07 | 0.421 |  | 0.45 | 0.16-1.22 | 0.142 |
| *KIR2DL4* | 98.6 | 100.0 | 100.0 | 100.0 |  | 0.00 | 0.00-NaN | 0.324 |  | - | - | - |  | - | - | - |
| *KIR2DL5* | 63.9 | 57.1 | 65.0 | 60.7 |  | 1.15 | 0.64-2.05 | 0.661 |  | 0.86 | 0.38-1.96 | 0.834 |  | 1.20 | 0.45-3.19 | 0.810 |
| *KIR2DS1* | 11.1 | 7.1 | 10.0 | 10.0 |  | 1.13 | 0.45-2.79 | 0.816 |  | 0.69 | 0.15-3.21 | 1.000 |  | 1.00 | 0.21-4.74 | 1.000 |
| *KIR2DS2* | 62.5 | 53.6 | 60.0 | 56.7 |  | 1.27 | 0.72-2.27 | 0.468 |  | 0.88 | 0.39-1.98 | 0.837 |  | 1.15 | 0.44-2.97 | 0.815 |
| *KIR2DS3* | 31.9 | 25.0 | 30.0 | 28.0 |  | 1.21 | 0.66-2.22 | 0.637 |  | 0.86 | 0.34-2.17 | 0.822 |  | 1.10 | 0.40-3.06 | 0.798 |
| *KIR2DS4* | 98.6 | 100.0 | 95.0 | 98.7 |  | 0.96 | 0.09-10.76 | 1.000 |  |  | NaN- | 1.000 |  | 0.26 | 0.02-2.97 | 0.315 |
| *KIR2DS5* | 43.1 | 35.7 | 40.0 | 43.3 |  | 0.99 | 0.56-1.74 | 1.000 |  | 0.73 | 0.31-1.68 | 0.534 |  | 0.87 | 0.34-2.26 | 0.815 |
| *KIR2DP1* | 95.8 | 96.4 | 90.0 | 98.7 |  | 0.31 | 0.05-1.90 | 0.332 |  | 0.36 | 0.03-4.17 | 0.403 |  | 0.12 | 0.02-0.92 | 0.068 |
| *KIR3DL1* | 98.6 | 100.0 | 95.0 | 99.3 |  | 0.48 | 0.03-7.73 | 0.544 |  |  | NaN- | 1.000 |  | 0.13 | 0.01-2.12 | 0.222 |
| *KIR3DL2* | 100.0 | 100.0 | 100.0 | 100.0 |  | - | - | - |  | - | - | - |  | - | - | - |
| *KIR3DL3* | 100.0 | 100.0 | 100.0 | 100.0 |  | - | - | - |  | - | - | - |  | - | - | - |
| *KIR3DS1* | 8.3 | 0.0 | 10.0 | 8.7 |  | 0.96 | 0.35-2.63 | 1.000 |  | 0.00 | 0.00-NaN | 0.226 |  | 1.17 | 0.24-5.62 | 0.691 |
| *KIR3DP1* | 100.0 | 100.0 | 100.0 | 100.0 |  | - | - | - |  | - | - | - |  | - | - | - |

*: total infected group of infants comprised of the IP and IU subsets as well as infected infants whose timing of infection could not be determined
