# Supplementary material for: KIR-HLA and Maternal-Infant HIV-1 Transmission in Sub-Saharan Africa
Source: PLoS One. 2011 Feb 4;6(2):e16541. doi: 10.1371/journal.pone.0016541 (PMC3035631; doi:10.1371/journal.pone.0016541)
Supplement: Table S3 — Comparison of frequencies of KIR3DL1, KIR3DS1 and HLA-Bw allotypes as well as KIR-HLA-Bw combinations between HIV-1-transmitting (TR) mothers, intrapartum (IP)-HIV-1-transmitting mothers, intrauterine (IU)-HIV-1-transmitting mothers and non-transmitting (NT) mothers. (DOC) [file pone.16541.s003.doc]

Table S3. Comparison of frequencies of *KIR3DL1*, *KIR3DS1* and *HLA-Bw* allotypes as well as *KIR-HLA-Bw* combinations between HIV-1-transmitting (TR) mothers, intrapartum (IP)-HIV-1-transmitting mothers, intrauterine (IU)-HIV-1-transmitting mothers and non-transmitting (NT) mothers

| **Genetic factor** | **TR**  **mothers**  **(N=72-74)** | **IP**  **mothers**  **(N=29)** | **IU**  **mothers**  **(N=21)** | **NT**  **mothers**  **(N=149-150)** |  | **TR mothers vs NT mothers** | | |  | **IP mothers vs NT mothers** | | |  | **IU mothers vs NT mothers** | | |
| --- | --- | --- | --- | --- | --- | --- | --- | --- | --- | --- | --- | --- | --- | --- | --- | --- |
|  | **% representation** | | | |  | **OR** | **95% CI** | ***P*** |  | **OR** | **95% CI** | ***P*** |  | **OR** | **95% CI** | ***P*** |
| ***KIR* alleles** |  |  |  |  |  |  |  |  |  |  |  |  |  |  |  |  |
| *3DL1/3DL1* | 94.6 | 93.1 | 95.2 | 94.7 |  | 0.99 | 0.29-3.39 | 1.000 |  | 0.76 | 0.15-3.78 | 0.666 |  | 1.13 | 0.13-9.49 | 1.000 |
| *3DL1/3DS1* | 4.1 | 6.9 | 0.0 | 4.7 |  | 0.86 | 0.22-3.44 | 1.000 |  | 1.51 | 0.30-7.68 | 0.640 |  | 0.00 | 0.00-NaN | 1.000 |
| *3DS1/3DS1* | 0.0 | 0.0 | 0.0 | 0.7 |  | 0.00 | 0.00-NaN | 1.000 |  | 0.00 | 0.00-NaN | 1.000 |  | 0.00 | 0.00-NaN | 1.000 |
| ***HLA-B* alleles** |  |  |  |  |  |  |  |  |  |  |  |  |  |  |  |  |
| *Bw4/Bw4* | 18.1 | 13.8 | 19.1 | 15.3 |  | 1.22 | 0.58-2.57 | 0.698 |  | 0.88 | 0.28-2.78 | 1.000 |  | 1.30 | 0.40-4.21 | 0.749 |
| *Bw4/Bw6* | 40.3 | 37.9 | 42.9 | 46.7 |  | 0.77 | 0.44-1.36 | 0.390 |  | 0.70 | 0.31-1.58 | 0.422 |  | 0.86 | 0.34-2.15 | 0.818 |
| *Bw6/Bw6* | 41.7 | 48.3 | 33.3 | 38.0 |  | 1.17 | 0.66-2.07 | 0.660 |  | 1.52 | 0.68-3.39 | 0.308 |  | 0.82 | 0.31-2.14 | 0.811 |
| ***KIR-HLA* combinations** |  |  |  |  |  |  |  |  |  |  |  |  |  |  |  |  |
| *3DL1* + *Bw4* | 58.9 | 51.7 | 66.7 | 61.3 |  | 0.90 | 0.51-1.60 | 0.771 |  | 0.68 | 0.30-1.50 | 0.409 |  | 1.26 | 0.48-3.31 | 0.811 |
| *3DL1*+*Bw480Ile* | 45.8 | 41.4 | 45.0 | 45.3 |  | 1.02 | 0.58-1.79 | 1.000 |  | 0.85 | 0.38-1.91 | 0.839 |  | 0.99 | 0.39-2.52 | 1.000 |
| *3DL1*004*+*Bw4* | 11.0 | 3.4 | 19.0 | 9.3 |  | 1.20 | 0.48-2.99 | 0.811 |  | 0.35 | 0.04-2.75 | 0.471 |  | 2.29 | 0.67-7.74 | 0.244 |
| *3DL1*004*+*Bw480Ile* | 8.3 | 3.4 | 10.0 | 5.3 |  | 1.61 | 0.54-4.84 | 0.390 |  | 0.63 | 0.08-5.27 | 1.000 |  | 1.97 | 0.39-10.02 | 0.333 |
| *3DS1*+*Bw4* | 1.4 | 0.0 | 0.0 | 2.7 |  | 0.51 | 0.06-4.62 | 1.000 |  | 0.00 | 0.00-NaN | 1.000 |  | 0.00 | 0.00-NaN | 1.000 |
| *3DS1*+*Bw480Ile* | 1.4 | 0.0 | 0.0 | 2.0 |  | 0.69 | 0.07-6.75 | 1.000 |  | 0.00 | 0.00-NaN | 1.000 |  | 0.00 | 0.00-NaN | 1.000 |
| *3DL1*+*Bw4Bw4* | 18.1 | 13.8 | 19.1 | 15.3 |  | 1.22 | 0.58-2.57 | 0.698 |  | 0.88 | 0.28-2.78 | 1.000 |  | 1.30 | 0.40-4.21 | 0.749 |
| *3DS1*+*Bw4Bw4* | 1.4 | 0.0 | 0.0 | 0.7 |  | 2.10 | 0.13-34.04 | 0.544 |  | 0.00 | 0.00-NaN | 1.000 |  | 0.00 | 0.00-NaN | 1.000 |
| *3DL1/3DL1*+*Bw4/Bw4* | 16.7 | 13.8 | 19.1 | 14.7 |  | 1.16 | 0.54-2.51 | 0.695 |  | 0.93 | 0.30-2.94 | 1.000 |  | 1.37 | 0.42-4.45 | 0.532 |
| *3DL1/3DL1*+*Bw4/Bw6* | 40.3 | 37.9 | 42.9 | 44.7 |  | 0.84 | 0.47-1.48 | 0.565 |  | 0.76 | 0.34-1.71 | 0.545 |  | 0.93 | 0.37-2.34 | 1.000 |
| *3DL1/3DL1*+*Bw6/Bw6* | 37.5 | 41.4 | 28.6 | 35.3 |  | 1.10 | 0.61-1.97 | 0.767 |  | 1.29 | 0.57-2.91 | 0.535 |  | 0.73 | 0.27-2.00 | 0.630 |
| *3DL1/3DS1*+*Bw4/Bw4* | 1.4 | 0.0 | 0.0 | 0.7 |  | 2.10 | 0.13-34.04 | 0.544 |  | 0.00 | 0.00-NaN | 1.000 |  | 0.00 | 0.00-NaN | 1.000 |
| *3DL1/3DS1*+*Bw4/Bw6* | 0.0 | 0.0 | 0.0 | 1.3 |  | 0.00 | 0.00-NaN | 1.000 |  | 0.00 | 0.00-NaN | 1.000 |  | 0.00 | 0.00-NaN | 1.000 |
| *3DL1/3DS1*+*Bw6/Bw6* | 2.8 | 6.9 | 0.0 | 2.7 |  | 1.04 | 0.19-5.83 | 1.000 |  | 2.70 | 0.47-15.50 | 0.251 |  | 0.00 | 0.00-NaN | 1.000 |
