# Supplementary material for: KIR-HLA and Maternal-Infant HIV-1 Transmission in Sub-Saharan Africa
Source: PLoS One. 2011 Feb 4;6(2):e16541. doi: 10.1371/journal.pone.0016541 (PMC3035631; doi:10.1371/journal.pone.0016541)
Supplement: Table S4 — Comparison of frequencies of KIR3DL1, KIR3DS1 and HLA-Bw allotypes as well as KIR-HLA-Bw combinations between HIV-1-infected (INF) infants, intrapartum (IP)-HIV-1-infected infants, intrauterine (IU)-HIV-1-infected infants and exposed uninfected (EU) infants. (DOC) [file pone.16541.s004.doc]

Table S4. Comparison of frequencies of KIR3DL1, KIR3DS1 and HLA-Bw allotypes as well as KIR-HLA-Bw combinations between HIV-1-infected (INF) infants, intrapartum (IP)-HIV-1-infected infants, intrauterine (IU)-HIV-1-infected infants and exposed uninfected (EU) infants

| **Genetic factor** | **INF**  **infants**  **(N=70-72)** | **IP**  **infants (N=28)** | **IU**  **infants**  **(N=20)** | **EU**  **infants**  **(N=149-150)** |  | **INF infants vs EU infants** | | |  | **IP infants vs EU infants** | | |  | **IU infants vs EU infants** | | |
| --- | --- | --- | --- | --- | --- | --- | --- | --- | --- | --- | --- | --- | --- | --- | --- | --- |
|  | **% representation** | | | |  | **OR** | **95% CI** | ***P*** |  | **OR** | **95% CI** | ***P*** |  | **OR** | **95% CI** | ***P*** |
| ***KIR* alleles** |  |  |  |  |  |  |  |  |  |  |  |  |  |  |  |  |
| *3DL1/3DL1* | 91.7 | 100.0 | 90.0 | 91.3 |  | 1.04 | 0.38-2.87 | 0.999 |  |  | NaN- | 0.226 |  | 0.85 | 0.18-4.10 | 0.691 |
| *3DL1/3DS1* | 6.9 | 0.0 | 5.0 | 8.0 |  | 0.86 | 0.29-2.54 | 0.999 |  | 0.00 | 0-NaN | 0.218 |  | 0.61 | 0.07-7.92 | 1.000 |
| *3DS1/3DS1* | 1.4 | 0.0 | 5.0 | 0.7 |  | 2.10 | 0.13-34.04 | 0.545 |  | 0.00 | 0-NaN | 1.000 |  | 7.84 | 0.47-130.61 | 0.222 |
| ***HLA-B* alleles** |  |  |  |  |  |  |  |  |  |  |  |  |  |  |  |  |
| *Bw4/Bw4* | 15.7 | 14.3 | 20.0 | 16.8 |  | 0.93 | 0.43-2.02 | 1.000 |  | 0.83 | 0.26-2.59 | 1.000 |  | 1.24 | 0.38-4.02 | 0.753 |
| *Bw4/Bw6* | 47.1 | 39.3 | 65.0 | 49.7 |  | 0.90 | 0.51-1.60 | 0.773 |  | 0.66 | 0.29-1.49 | 0.410 |  | 1.88 | 0.71-4.98 | 0.238 |
| *Bw6/Bw6* | 37.1 | 46.4 | 15.0 | 33.6 |  | 1.17 | 0.65-2.12 | 0.649 |  | 1.72 | 0.76-3.88 | 0.203 |  | 0.35 | 0.10-1.25 | 0.124 |
| ***KIR-HLA* combinations** |  |  |  |  |  |  |  |  |  |  |  |  |  |  |  |  |
| *3DL1* + *Bw4* | 62.5 | 53.6 | 80.0 | 65.3 |  | 0.87 | 0.48-1.56 | 0.654 |  | 0.61 | 0.27-1.38 | 0.286 |  | 2.12 | 0.67-6.68 | 0.217 |
| *3DL1*+*Bw480Ile* | 43.1 | 28.6 | 55.0 | 49.3 |  | 0.77 | 0.44-1.35 | 0.390 |  | 0.41 | 0.17-0.99 | **0.062** |  | 1.26 | 0.49-3.20 | 0.812 |
| *3DL1*004*+*Bw4* | 8.3 | 10.7 | 10.0 | 11.4 |  | 0.71 | 0.27-1.87 | 0.639 |  | 0.93 | 0.25-3.42 | 1.000 |  | 0.86 | 0.18-4.05 | 1.000 |
| *3DL1*004*+*Bw480Ile* | 5.6 | 3.6 | 10.0 | 8.1 |  | 0.67 | 0.21-2.16 | 0.590 |  | 0.42 | 0.05-3.39 | 0.695 |  | 1.27 | 0.26-6.13 | 0.673 |
| *3DS1*+*Bw4* | 6.9 | 0.0 | 10.0 | 6.7 |  | 1.04 | 0.34-3.16 | 0.999 |  | 0.00 | 0-NaN | 0.366 |  | 1.56 | 0.32-7.67 | 0.636 |
| *3DS1*+*Bw480Ile* | 6.9 | 0.0 | 10.0 | 4.7 |  | 1.51 | 0.46-4.95 | 0.533 |  | 0.00 | 0-NaN | 0.598 |  | 0.44 | 0.08-2.29 | 0.286 |
| *3DL1*+*Bw4Bw4* | 15.7 | 14.3 | 20.0 | 16.7 |  | 0.93 | 0.43-2.02 | 1.000 |  | 0.83 | 0.27-2.61 | 1.000 |  | 1.25 | 0.39-4.06 | 0.752 |
| *3DS1*+*Bw4Bw4* | 2.9 | 0.0 | 5.0 | 0.7 |  | 4.38 | 0.39-49.16 | 0.238 |  | 0.00 | 0-NaN | 1.000 |  | 7.84 | 0.47-130.61 | 0.222 |
| *3DL1/3DL1*+*Bw4/Bw4* | 12.9 | 14.3 | 15.0 | 16.1 |  | 0.77 | 0.34-1.75 | 0.686 |  | 0.87 | 0.28-2.73 | 1.000 |  | 0.92 | 0.25-3.38 | 1.000 |
| *3DL1/3DL1*+*Bw4/Bw6* | 42.9 | 39.3 | 60.0 | 43.6 |  | 0.97 | 0.55-1.72 | 1.000 |  | 0.84 | 0.37-1.91 | 0.836 |  | 1.94 | 0.75-5.02 | 0.232 |
| *3DL1/3DL1*+*Bw6/Bw6* | 35.7 | 46.4 | 15.0 | 31.5 |  | 1.21 | 0.66-2.19 | 0.542 |  | 1.88 | 0.83-4.27 | 0.134 |  | 0.38 | 0.12-1.37 | 0.191 |
| *3DL1/3DS1*+*Bw4/Bw4* | 2.9 | 0.0 | 5.0 | 0.7 |  | 4.35 | 0.39-48.84 | 0.240 |  | 0.00 | 0-NaN | 1.000 |  | 7.79 | 0.47-129.73 | 0.223 |
| *3DL1/3DS1*+*Bw4/Bw6* | 2.9 | 0.0 | 0.0 | 5.4 |  | 0.52 | 0.11-2.51 | 0.508 |  | 0.00 | 0-NaN | 0.359 |  | 0.00 | 0-NaN | 0.598 |
| *3DL1/3DS1*+*Bw6/Bw6* | 1.4 | 0.0 | 0.0 | 2.0 |  | 0.71 | 0.07-6.90 | 0.999 |  | 0.00 | 0-NaN | 1.000 |  | 0.00 | 0-NaN | 1.000 |

Bold *P* values indicate trends (0.05<*P*<0.1) or significant differences (*P*<0.05)
